# Electron transport (0.2 eV–10 keV) in liquid water: resolving discrepancies between track simulations and radiolysis data

**DOI:** 10.1039/d6ra00710d

**Published:** 2026-04-17

**Authors:** Hoon Lee, David M. Bartels, Ryan G. McClarren

**Affiliations:** a Notre Dame Radiation Laboratory, University of Notre Dame Notre Dame Indiana 46556 USA rmcclarr@nd.edu; b Department of Aerospace and Mechanical Engineering, University of Notre Dame Notre Dame Indiana 46556 USA; c Department of Chemistry and Biochemistry, University of Notre Dame Notre Dame Indiana 46556 USA

## Abstract

The scattering and transport processes of electrons with initial kinetic energies ranging from 0.2 eV to 10 keV in liquid-phase water are studied using a Monte Carlo (MC) simulation. This study aims to identify a set of scattering cross sections and physical assumptions regarding angular deflection and energy loss that ensure computational results align with available data across the entire energy range of interest. These data include thermalization distances derived from photoinjection measurements in the very low energy regime (<2.5 eV) and theoretical approximations, including continuously slowing down approximation (CSDA) values, up to 10 keV. In particular, incorporating an assumption for track termination events—such as transient negative anion (TNA) formation at resonance peaks followed by dissociative electron attachment (DEA) processes—may resolve the longstanding discrepancy in geminate separation distance of secondary electrons, which has been reported to vary over the rather broad range of 6–14 nm. This discrepancy arises between stochastic models fitted to diffusive spur recombination data from radiolysis measurements and those directly calculated using MC track simulations with measured scattering cross sections. By accounting for secondary electron tracks and electron autodetachment (EAD) from TNA states, as well as autoionization of neutral excited water molecules, the model also reproduces the reported *G*-value of pre-solvated electrons.

## Abstract 2

*E*_0_-dependent electron geminate separation distances over the range of 0.2 eV to 10 keV.

## Graphical abstract

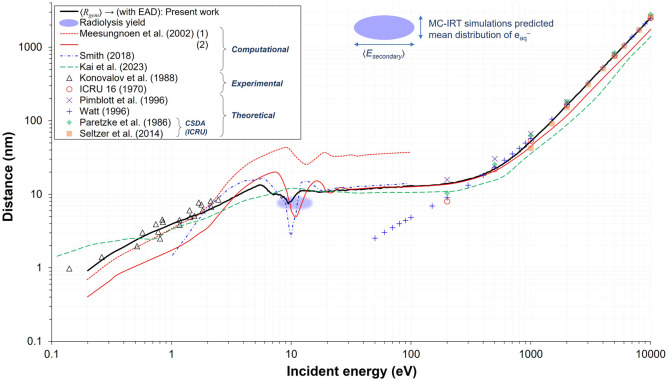

## 1. Introduction

Scattering processes of energetic particles in irradiated media, ranging from low-temperature ices to supercritical fluids, have been studied extensively over the past several decades.^1,2^ In addition to photon energy from absorption of X-rays or Compton scattering of gamma-rays, ionizing radiation can also be delivered by particles such as α, β, or heavy ions. As these particles decelerate along their path, they generate numerous secondary electrons (also known as *δ*-rays or knock-on electrons) from the molecules that constitute the medium. Understanding how these secondary electrons interact with condensed matter in radiolysis is fundamental to various scientific and technological fields, including radiation therapy, space radiation protection, and nuclear engineering.^3–5^

In general, high energy electrons lose their energy through a sequence of inelastic electronic interactions, predominantly ionization at high energies, with electronic excitation becoming increasingly important at lower energies.^6^ Once their energy has degraded below the band gap of the material, energy is lost *via* vibrational and rotational excitations, until ultimately the electrons are “thermalized” and/or trapped. The energy deposition process is dubbed the physical stage of radiolysis, lasting perhaps 100 femtoseconds. This is followed by the physico-chemical stage, lasting perhaps a picosecond, in which the deposited energy is dissipated, often by breaking chemical bonds or ionizing molecules. Some fraction of the fragmented molecules, liberated electrons and “holes” will then recombine in the chemical stage, lasting from hundreds of nanoseconds to microseconds in a typical liquid. Those reactive species which survive the chemical stage can then do other chemistry in the “bulk” of the material.^7^

Naturally water is the most important substance to understand given its presence in living matter as well as its technological use in high-radiation environments for cooling and as a solvent. Most of our understanding of water radiolysis comes from chemical scavenging and spectroscopic studies employing advanced measurement technologies.^8^ While the chemical stage is relatively well established, the preceding physical and physico-chemical stages remain subjects of ongoing debate, with many aspects yet to reach consensus. Key challenges include constructing a rigorous set of electron scattering cross sections that quantify the probabilities of scattering energy into vibrational, rotational, or electronic degrees of freedom; elucidating the associated energy losses and angular deflections for various collision events; and determining the initial energy spectra of secondary electrons. Extensive data are available for gas-phase water^9–15^ and amorphous solid water (ASW);^16–21^ however, data for liquid-phase water remain limited.^1,2,22–27^

ASW, characterized by its non-crystalline structure, represents the predominant form of water in the universe.^28^ The ASW electron scattering cross section data sets currently available^21^ are from measurements on low-density amorphous (LDA) ice, as reported by Sanche and coworkers. From an optical standpoint, the dipole oscillator strength distribution (DOSD) of ASW also appears nearly identical to that of liquid water.^29^ Signorell recently emphasized the close resemblance between ASW and liquid water in terms of electron scattering behavior.^30^ Many recent studies, including our previous work,^31^ have utilized and validated these ASW cross section data sets,^32–35^ despite their origin more than two decades ago; their continued use underscores the robustness of the data. Nevertheless, simulation results for key transport properties—such as thermalization distance, *R*_th_, inelastic mean free path (IMFP) and elastic mean free paths (EMFP), and low energy scattering probabilities—derived from models using ASW-based cross sections deviate significantly from experimental results for liquid water. These discrepancies become increasingly pronounced at lower energies,^34,36–39^ raising critical questions about the most appropriate data sets for investigating liquid-phase water. Such differences are too large to be solely attributed to density variations between ASW and liquid water and remain without a definitive explanation.

Computer simulations provide a means to overcome experimental limitations. Since their first application focused on evaluating the range and straggling of high energy electrons,^40^ Monte Carlo (MC) methods have been widely adopted in various computational studies as a primary tool for calculating track structure, transport properties, and reactions associated with charged particles' scattering and energy deposition events due to their stochastic and probabilistic nature.^41–47^ However, despite significant advancements—such as the development of high-fidelity models like GEANT4 and TOPAS^48–50^—large uncertainties and substantial computational demands remain major obstacles to fully assessing the strengths and limitations of these tools. This challenge is further compounded by scarcity and inconsistency of available measurements, making it difficult to provide reliable input parameters for modeling and to validate the resulting simulations against experiment.^51^

The secondary electrons ejected from a water molecule with an initial kinetic energy in the several-eV range or, at most, a few tens of eV, rapidly (in hundreds of femtoseconds) lose their kinetic energy. The distance these low energy electrons (LEE) travel relative to their origin at the ionization core before becoming hydrated (*i.e.*, *R*_th_) is critical, as it largely determines the probability of subsequent diffusive recombination during the chemical and biological stages of water radiolysis. A subset of these electrons may be temporarily captured by water molecules and subsequently ejected; in such cases, the relevant characteristic length scale is the ejection length. The superposition of these two distinct distances (*i.e.*, geminate separation distance) represents the initial spatial distribution of solvated electrons (e_aq_^−^), when averaged over the initial energy spectrum of secondary electrons. Probability of diffusive recombination of an electron with its geminate partner(s) depends mainly on their initial separation.^52–55^ Therefore, the initial spatial distribution between e_aq_^−^ and geminate radicals, determined during the physico-chemical stage, is perhaps the most significant parameter influencing the outcome of radiation-initiated chemistry, because e_aq_^−^ itself is one of the most important reactive intermediates in water radiolysis, driving many of the reduction reactions in chemical and biological stages.

In our previous study,^31^ we demonstrated a MC model that successfully reproduced the only available electron energy loss spectroscopy (EELS) spectra of ASW films deposited on a platinum substrate (Pt–ASW EELS) for two LEE cases (14.3 and 19 eV),^56^ employing ASW cross sections determined from the corresponding EELS experiments.^17–19,21^

Building upon this previous MC framework, the present work extends and refines the model to describe transport characteristics—energy loss, angular deflection, and thermalization—of electrons in liquid water with improved physical accuracy and broader energy coverage. The objective of this work is to identify the key modeling parameters and physical assumptions that enable the model to accurately reproduce experimental data spanning 0.2 eV to 10 keV and to reconcile discrepancies in geminate separation distance predicted by different methodologies. The energy range is chosen to be low enough to allow meaningful comparison with direct photoinjection measurements,^57^ while remaining high enough to ensure reliability through validation against theoretical approximations, including continuously slowing-down approximation (CSDA) data sets, reported in previous studies.^41,58–61^ Because experimental data are limited—and those available, such as the ASW cross sections^21^ and DOSD data,^29,62,63^ cover only the approximate range from 1–2 eV up to 100–170 eV—careful and physically consistent extrapolation is essential to maintain physical plausibility and continuity across the full energy range of interest. To construct a robust set of cross sections that can be compared with existing computational frameworks, including event-by-event track-structure implementations such as the nBio extension and GEANT4-DNA physics model used within OpenTOPAS v4.0 (based on GEANT4 v11),^46,64–67^ we adopted the experimentally validated EMFP and the computationally obtained angular differential cross section (ADCS) for elastic scattering from a recent study of Gadeyne *et al.*,^68^ and incorporated a cross section for track termination events due to transient negative anion (TNA) formation, scaled from recommended gas-phase data.^14^ Additionally, we established an ionization energy loss sampling scheme based on binary collision kinematics and a dielectric energy loss function (ELF) derived from DOSD data, representing electronic energy loss for incident energies above 100 eV. For electrons with incident energies 100 eV and below, where the role of momentum transfer becomes increasingly important, we employed the single differential cross section (SDCS), also referred to as the energy loss distribution function,^69,70^ as in our previous study, to sample the electronic energy loss. We further investigated the impact of various elastic cross section data sets and the presence of TNA resonances on reproducing the average spur radius inferred from a combination of statistical analyses and picosecond radiolysis measurements. Finally, the initial *G*-value of pre-solvated electron was determined by accounting for all secondary electron tracks, including electrons ejected through the TNA-driven electron autodetachment (EAD) pathway and additional electrons generated *via* autoionization of neutral excited water molecules. This comprehensive analysis elucidates the complexities of electron–matter interactions in condensed phases and provides insights into improving the accuracy of spatial extent of the e_aq_^−^ distribution in radiation chemistry modeling.

## 2. Modeling methods

**Fig. 1:**
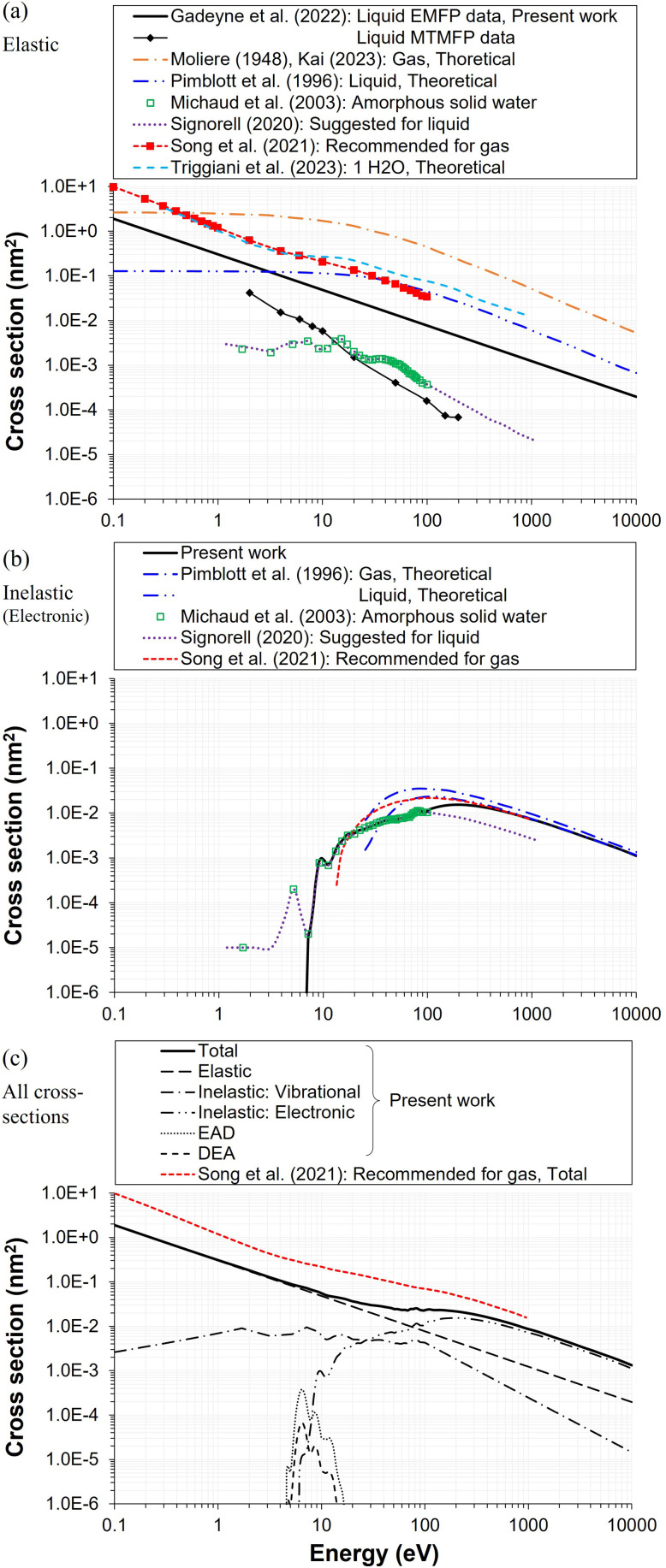
Electron scattering cross sections for liquid-phase water used in this study, compared with selected values used in the literature. (a) Elastic cross sections reported in various studies, including theoretical values,^9,15,41,47^ the original^21^ and extended^30^ ASW data sets, recommended gas-phase data,^14^ and extrapolated values suggested for liquid water derived from experimental and *ab initio* calculations of EMFP and MTMFP.^68^ (b) Electronic inelastic cross sections reported in various studies, including theoretical values,^41^ original^21^ and extended^30^ ASW data sets, and recommended gas-phase data.^14^ (c) Compiled data sets including vibrational inelastic events, electron autodetachment (EAD) and dissociative electron attachment (DEA). The total scattering cross section from the recommended gas-phase data^14^ is shown for comparison to illustrate the difference between phases.

Throughout this work, an electron is modeled as a particle with mass injected into a medium of uniform density, liquid-phase water at room temperature, with an initial kinetic energy. The electron is tracked ballistically until it is either captured or slows down to an energy below the cutoff threshold of 0.2 eV, at which point it is considered thermalized. Although 0.2 eV lies above the thermal energy at room temperature (*k*_B_*T*), electron transport in the sub-eV regime is dominated by elastic scattering. In this energy range, large-angle scattering becomes significant, leading to diffusive transport governed by the momentum transfer mean free path (MTMFP), which becomes comparable to the EMFP as shown later in Fig. 1 (c). As a result, electron motion in the sub-eV regime resembles a true random walk with very small step lengths and contributes only weakly to the net displacement. Moreover, the present simulations treat electrons as classical particles and do not explicitly include wave effects, for which the physical validity of a classical description becomes increasingly questionable below ∼0.1–0.2 eV. The cutoff energy adopted here therefore represents a practical termination criterion, consistent with previous track-structure simulations employing the same^71^ and a similar treatment (0.1 eV),^35,72^ rather than a physical definition of thermal equilibrium.

Upon capture, the electron loses all its kinetic energy, and the track is terminated. The transport process of an energetic particle is inherently stochastic, necessitating the use of variables governed by specific probability density functions (PDFs). In radiation chemistry, Poisson and exponential distributions are commonly employed in the continuum method of MC simulation to describe the occurrence of rare events over a fixed period. In this context, random numbers are used to sample propagation (jump) distances between successive events which are determined by macroscopic (integrated) cross sections at a given energy. Once the separation distance is known, the time until the next event can be calculated, along with the traveling distance and *R*_th_ from the origin to the endpoint of the track.

### 2.1. Cross sections

In stochastic electron-track modeling, the frequency and nature of collisions are determined by the individual and total scattering cross sections of the medium. Fig. 1 (a–c) presents the compiled cross section data sets used for this study, together with relevant data from previous references for each collision type to facilitate comparison.

#### 2.1.1 Inelastic

The inelastic cross sections used in this study—both vibrational and electronic—are based on the ASW data sets of Sanche and coworkers,^21^ derived from Pt–ASW EELS experiments utilizing a two-stream multiple scattering analysis.^18,19^ This analysis involves numerically fitting the data to determine the relative magnitudes of the major energy loss channels, and converting the normalized incident beam current into a double differential scattering cross section, *d*^2^*σ*/d*Ω*d*E*, while accounting for multiple-scattering effects and the thickness dependence of elastically backscattered intensity to determine the total scattering probability.^17^ By analyzing the energy distribution of backscattered electrons (*i.e.*, energy loss spectra) from the ASW film composed of multiple condensed layers, the method enables the separation of contributions from elastic and various inelastic scattering processes. This, in turn, allows for the quantification of the elastic scattering probability and the determination of the integral elastic cross section.

#### 2.1.2 Elastic

The elastic cross section is one of the key parameters in calculating *R*_th_. It does not involve energy loss but plays a dominant role in determining electron transport and overall spread, particularly in the low energy region, where elastic scattering predominates and remains large, unlike the inelastic cross section, which decreases toward zero as shown in Fig. 1 (c). As a result, elastic scattering critically influences the average spur size in radiolysis.^73^ Depending on its angular deflection, characterized by the anisotropy factor^21^ or by the ADCS data,^68^ electron trajectories are more directional relative to purely isotropic scattering. Therefore, the accuracy of the elastic cross section directly affects the reliability of MC track simulations. Reported total elastic and momentum transfer cross sections, which weight elastic scattering by 1 − cos *θ* and therefore quantify the effectiveness of momentum randomization, differ dramatically within both the gas- and liquid-phases of water,^14,68^ with momentum transfer cross sections appearing smaller than the corresponding elastic cross sections by factors ranging from approximately 3–4 up to nearly an order of magnitude in the 1–100 eV energy range in both phases, due to the dominance of forward-peaked scattering events. In the Pt–ASW EELS experiments used to derive the ASW cross sections,^17–19,21^ forward-peaked anisotropic events were not accounted for because they do not contribute to beam attenuation.^21,30^ Consequently, the reported ASW “elastic” cross section effectively corresponds to the momentum transfer cross section.^30,68^ The gas-phase data shown in Fig. 1 (a)^9,15,47^ likewise raise questions about their applicability to liquid-phase systems,^74^ as the calculated EMFP at liquid density becomes smaller than the average intermolecular distance (0.31 nm, corresponding to approximately 0.1 nm^2^ of cross section in Fig. 1 (a)) not only in the low energy domain but even up to several hundred eV. Therefore, in this work, elastic cross sections derived from EMFP obtained in recent liquid microjet measurements and *ab initio* calculations^68^ were adopted to account for cumulative small-angle deflection effects that, though individually minor, may become significant when integrated over multiple scattering events.

#### 2.1.3 TNA-driven channels

Much of the academic literature emphasizes that successful simulation of LEE transport in condensed phase requires not only an understanding of the kinematics of the collision–reaction processes and energy–momentum balances that shape electron track trajectories, but also insight into electron trapping and capture mechanisms. Unfortunately, a molecular-level understanding of these processes in aqueous solutions, particularly in liquid water, remains limited. This lack of understanding has led to persistent discrepancies between stochastic modeling approaches, such as the independent reaction time (IRT) method^52,53^ used to interpret picosecond radiolysis spur measurements, and direct MC track simulations that estimate the 〈*R*_th_〉 of LEEs.^34,36^ These discrepancies suggest that crucial aspects of the thermalization process may have been omitted or overlooked due to the non-classical behavior of LEEs, substantial uncertainties in experimental measurements, or a combination of both. This is particularly evident in the lack of detailed insights into TNA resonances at energies around 5–15 eV.^23,24,27,75,76^ These short-lived anionic states, formed when a LEE is temporarily captured by a water molecule or molecular cluster in an unstable orbital, may play a key role in determining the fate of LEEs in condensed media. TNA resonances serve as gateways to competing decay channels, most notably dissociative electron attachment (DEA) and EAD.^77^ In DEA, the captured electron induces bond cleavage and becomes chemically bound to the dissociating fragment, thereby terminating the track. In the case of EAD in condensed media, although the electron is not permanently bound, the decay proceeds through rapid relaxation of a locally excited molecular state, and the electron is re-emitted only over very short length scales due to strong polarization and localization effects. As a result, EAD does not lead to the formation of a freely propagating subexcitation electron in the conduction band but instead effectively acts as a localized energy-loss and track-termination event, similar in outcome to trapping or autoionization processes. Consequently, both DEA and EAD channels shorten the average geminate separation distance (*i.e.*, weighted combination of 〈*R*_th_〉 and ejection length), albeit through distinct physical mechanisms.^10,78–80^ As conventional EELS studies^21,56^ are inherently unable to detect such track terminating or shortening events, gaps in understanding may not solely arise from measurement uncertainties (L. Sanche, personal communication, September 7, 2022). Consequently, modelers often need to postulate mechanisms and adjust data sets to reconcile discrepancies and address gaps left by incomplete or unavailable data.

From this perspective, one adjustment made to the original ASW cross section data sets was the removal of the lowest “peak” (a single data point) reported at approximately 5.2 eV (see Fig. 1 (b)) in the “Others” energy loss category, which the original authors attributed to DEA^21^—by which they must mean TNA formation. While no direct measurements of TNA cross sections exist for condensed water, substantial evidence indicates that DEA can occur in the condensed phase, leading to the formation of H^−^ ions and OH radicals, even though its frequency differs considerably from gas-phase observations.^16,20,47,81,82^ In the liquid-phase, however, direct experiments are extremely rare or virtually nonexistent. Consequently, many researchers have inferred that DEA is essentially absent in liquid water,^47,68,73,83^ owing both to the lack of experimental evidence and to theoretical predictions that ultrafast solvation and intermolecular relaxation rapidly quench TNA resonances in liquid water at ambient conditions.^1^ The gas-phase DEA cross section exhibits three resonances corresponding to H^−^, O^−^, and OH^−^ channels associated with distinct valence-excited states. Our simulations simply scale down the recommended gas-phase DEA cross section^14^ by a factor of 7 to achieve agreement between the model results and radiolysis yield required by experiment and IRT simulations (as shown later in Fig. 7 (b)).^52,53^ The chosen scaling factor makes the resulting cross section lower than in the gas-phase, but the factor itself is approximately three times higher than the reduction factor (1/20) proposed in a recent study of Kai *et al.*^47^ It should be recognized that the gas-phase data used as the reference for our scaling are more recent (*i.e.*, 1972^84^ for^47^*vs.* 2007^11^ for present work) and trusted.^14^ It is also worth noting that some experimental studies^16,20^ have reported that not only the intensity but also location, and number of resonance peaks differ between gas and condensed phases. However, the physical explanation for this difference remains unclear, therefore, we do not consider it in this work. Based on this approach, an additional cross section for EAD processes was postulated to account for autodetachment events, referencing studies that quantified the proportion of TNA leading to EAD processes.^85^

#### 2.1.4 Cross section extrapolation

It should also be emphasized that the ASW cross section data have inherent limitations, most notably their narrow energy range, with no data available below 1.7 eV or above 100 eV. Even within the accessible range, extensive interpolation must be applied, particularly below 10 eV, where only five data points exist.^21^ Considering that the energy range reported for 〈*R*_th_〉 from direct photoinjection measurements (0.15–2.5 eV)^57,86^ and for the theoretical approximations, including CSDA data sets (above 200 eV),^41,58–61^ lies largely outside this interval, this represents a critical constraint. While a recent study proposed refined data sets for liquid water based on ASW data—extending the inelastic (vibrational) cross section range down to 10^−4^ eV^73^—the data sets continuity was achieved by scaling ASW or gas-phase data sets in regions lacking measurements and connecting them smoothly with the existing data points. A similar approach was adopted in another recent work that extended the ASW cross sections up to approximately 1 keV,^30^ where the sub-7 eV region was slightly adjusted to fit experimental droplet velocity map images (VMI) results. Apart from this low energy correction, however, the ASW data sets were used in that study largely unchanged across most of the energy range, while extrapolation beyond 100 eV was performed by proportionally scaling either reported theoretical data (for electronic events)^87^ or gas-phase data (for elastic and vibrational processes)^88^ simply to match the ASW data at 100 eV. These refinements remain insufficient to fully cover the energy range of interest in this work. Moreover, when applying such extrapolation procedures, physical soundness can only be ensured by verifying that the adjusted data reproduce available experimental observations, rather than by merely extending the data sets through smooth continuation. Although phase-dependent differences in physico-chemical properties may diminish at sufficiently high energies, it is very rare that such relationships can be accurately represented by a simple scaling regardless of energy.

Accordingly, in this work, extrapolation was performed with an emphasis on not only maintaining physical plausibility and continuity but also ensuring consistency with available experimental results. For elastic scattering, the extrapolation was guided by a previous study,^68^ which reported that a rising trend of cross section toward the 0 eV limit provides better agreement with photoelectron spectra. For inelastic (vibrational) scattering, the cross section was extrapolated to decrease toward the 0 eV limit,^47,68,73^ with the maximum positioned near 1 eV to achieve consistency with direct photoinjection data below 2.5 eV.^57^ For electronic excitation and ionization events, interpolation was carried out using a mathematical fitting procedure that ensures a smooth connection between datasets: from the ASW data at 100 eV, to the recommended gas-phase values at 1 keV,^14^ and finally to the theoretical estimates for liquid water at 10 keV.^41^ In this high energy regime, differences between gas- and liquid-phase cross sections become negligible, consistent with the expectations from the oscillator strength sum rule.^47,89^ This is further supported by the fact that, at 1 keV, the recommended gas-phase values and the theoretical estimates for liquid water are essentially identical as shown in Fig. 1 (b).

### 2.2. Energy loss

#### 2.2.1 Inelastic (vibrational)

The amount of electron kinetic energy lost varies depending on the type of scattering. For inelastic collisions that excite water molecules into vibrational, librational, and rotational modes, the ASW values reported by Sanche and coworkers^21^ for each inferior component were used. This approach directly reflects the fact that the energy loss of very low energy (particularly for < 1 eV) electron is quantized into actual vibrational modes. Although some studies have sampled the energy loss from a single ELF or from incident-energy–dependent SDCS obtained using dielectric-based models,^47,68^ the ASW cross sections employed here are directly derived from experimental EELS measurements. In the sub-eV regime, where energy losses are known to occur *via* discrete vibrational excitations rather than a continuous spectrum, sampling from experimentally resolved phonon, librational, bending, and stretching bands avoids the need for continuum approximations inherent to dielectric-based models.

In these low energy events, the molecular excitation energy losses are below 1 eV, with the most probable energy loss being less than 0.1 eV (see Fig. S1 in the SI). Therefore, from the viewpoint of total energy dissipation, such low energy losses constitute only a minor portion of the overall energy deposition and become negligible as the incident energy increases due to the low cross section value (see Fig. 1 (c)). However, their contribution becomes very important in the subexcitation energy region (*i.e.*, below band gap energy, *E*_g_),^68^ where these LEEs dominate the transport and thermalization processes. As noted in the Introduction, the average energy of secondary electrons lies in the several-eV range, with the most probable energy around 1 eV (see Fig. S7 in the SI). These low energy interactions can therefore be regarded as a major factor influencing the agreement in 〈*R*_th_〉 between the model and the measured direct-photoinjection data reported for the 0.15–2.5 eV range.^57,86^

#### 2.2.2 Ionization (above 100 eV)

In contrast to the vibrational excitation events, electronic excitation events become significant only above their respective thresholds, yet once induced, they account for the dominant portion of the total energy loss during thermalization. Unlike the low energy regime—where large-angle scattering produces substantial momentum transfer due to the strong influence of the molecular interaction potential—the scattering becomes increasingly forward-directed as the incident energy increases, leading to a rapid decrease in net momentum transfer. For this reason, the total energy loss associated with electronic events was sampled separately in the simulations, with a boundary set at an incident energy of 100 eV, where the momentum transfer becomes practically insignificant (see Fig. 1 (a)). This choice of a 100 eV boundary is also consistent with radiolysis considerations, as the energy region most relevant to early-time chemistry lies below 100 eV and the secondary electron entry spectrum is consistently found to fall within this range for all distributions examined (see Fig. S7 in the SI). Moreover, because we use the ASW inelastic (electronic) cross section data without modification in this range, the ∼100 eV upper limit of that data set naturally provides an additional analysis threshold, aligning with the definition of LEE (<100 eV) introduced by Sanche and coworkers in ref. 21.

For incident electron energies above 100 eV, the ionization energy loss was sampled from a dielectric ELF derived from the DOSD^29^ and NIST data sets,^90^ which provides the optical properties, dielectric response, and atomic form factor of water. Since the inelastic X-ray scattering (IXS) spectra measured for DOSD data are only available up to 100–170 eV,^29,63^ extrapolation was applied up to 10 keV by fitting the data with an exponential decay function, ensuring that

1 *f*_2_ = Energy × oscillator strength

**Fig. 2:**
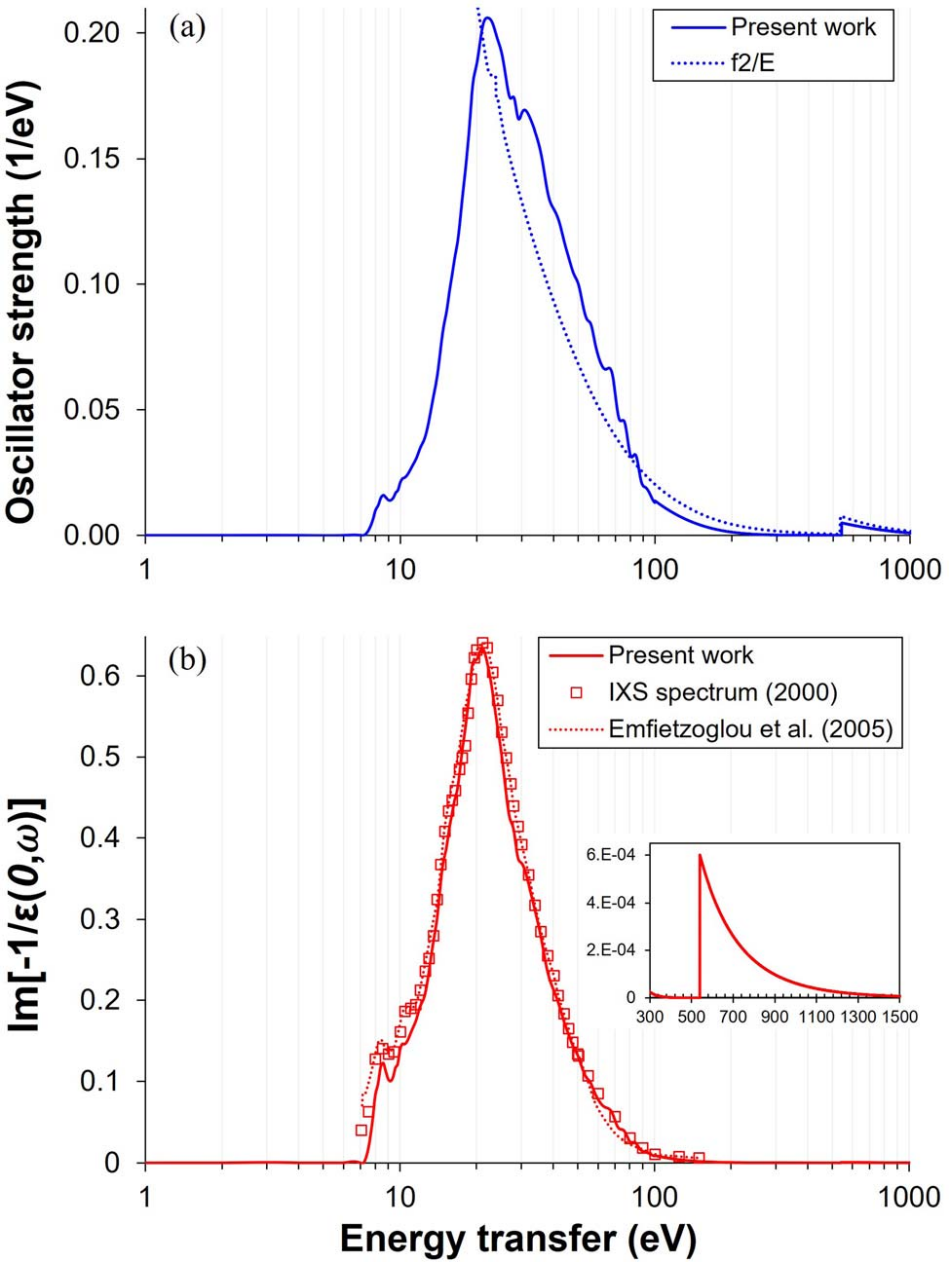
(a) DOSD: The solid line represents the constructed data sets from this work, based on reference data.^29^ The dotted line represents the oscillator strength derived from the atomic form factor, *f*_2_, as provided in the NIST data sets.^90^ The difference between the two arises from high uncertainties at low X-ray energy (<1 keV).^93^ (b) OELF: The solid line represents data directly derived from this work based on the DOSD data and IXS measurements.^29^ The dotted line illustrates the optical loss function of oxygen K-shell electrons, calculated using the NIST data sets^90^ and employing Drude model.^94,95^ Inset: a focus on the small O 1 s contribution from the K-shell.

2
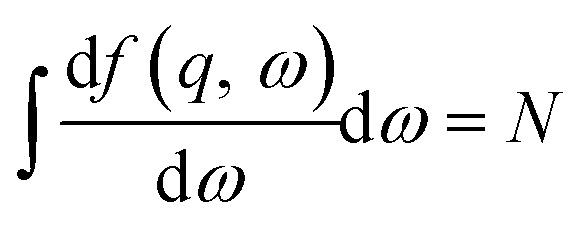
where *f*_2_ is the imaginary component of the atomic form factor, which primarily represents the absorption of X-rays by an atom at a specific energy, whereas *f*(*q*,*ω*)represents the generalized oscillator strength (GOS) as a function of momentum transfer, *q*, and energy transfer *ω*. The theoretical value of *N*, the total effective number of valence electrons, has been reported as 8.22–8.34,^29,62,63^ with small estimated corrections for multiple scattering within experimental uncertainties included (H. Hayashi, personal communication, February 1, 2024). It should be noted, however, that the O 1 s contribution of the K-shell must also be contained in the simulations by integrating the DOSD up to 10 keV, resulting in *N* becoming the total number of electrons in H_2_O (*i.e.*, 10). In this work, we obtained a value of 9.81 for *N*. The difference reflects the residual error associated with the fitted DOSD. The ELF can then be deduced using relationship between GOS and the dynamic structure factor, *S*,^62^ as,3
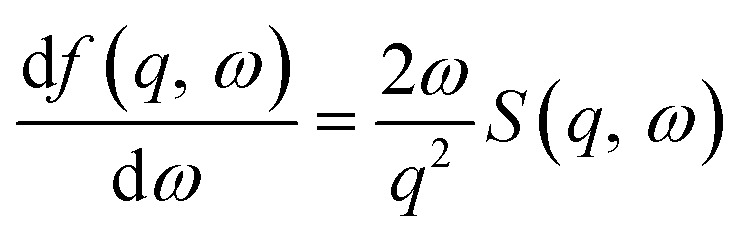
4
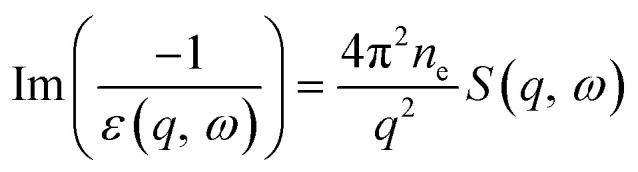
where *ε* is the dielectric response function, and *n*_e_ is the average electron density (∼3.34 × 10^23^/cm^3^). The imaginary part of the inverse dielectric response function, shown on the left-hand side of eqn (4), represents the ELF. In the optical limit, the ELF represents the dielectric response measured under negligible momentum transfer (*i.e.*, *q* → 0), as in optical reflection or absorption spectroscopy. Therefore, the optical ELF (OELF) corresponds to Im[−1/*ε*(0, *ω*)], which serves as the fundamental basis for constructing the extended ELF at finite momentum transfers. Fig. 2 (a and b) schematically illustrates the DOSD and the OELF used in this study, highlighting that the most probable energy loss in a single event by a fast (*i.e.*, low-linear energy transfer (LET)) electron is 22–23 eV, as reported in earlier studies.^41,91,92^ Reference data are also included for comparison.

The upper limit of ionization energy loss for an electron at a specific kinetic energy is determined based on the principles of conservation of energy and momentum.^96^ While some studies^35,41,92^ have assumed the maximum energy transfer to be 50–75% of the incident kinetic energy—thereby tracking the faster particle (*i.e.*, the one with higher energy) after ionization—this approach does not accurately determine the initial energy spectrum of emerging (secondary) electrons, which is essential for predicting the yield of e_aq_^−^. This is because the assumption arises purely from a tracking-algorithm convention introduced to resolve electron indistinguishability or mathematical partition points rather than from any underlying physical constraint. Although such events occur with very low probability, an electron can, in principle, lose nearly all its kinetic energy. In our calculations, the binding energy of the valence electron, representing ionization threshold, is directly sampled first from experimental photoelectron density of state (DOS) data (H. J. Wörner, personal communication, August 30, 2024). The energy of the primary electron after ionization and its scattering angle are then determined using classical scattering kinematics formulae relating angle-energy distribution to energy loss properties, namely^96^5
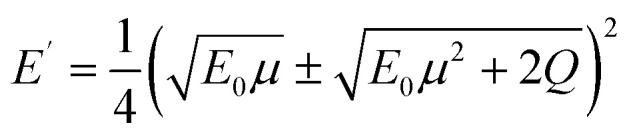
6
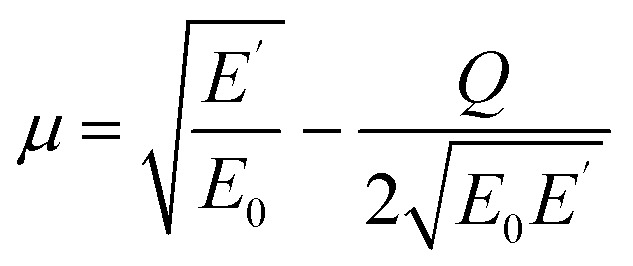
where *E*_0_ and *E*′ are the initial (incident) and final kinetic energies of the primary electrons, and *µ* is the scattering cosine. *Q* denotes the net gain or loss in kinetic energy of the products after an inelastic collision (0 if elastic) originally defined in the context of exo- or endoergic binary reactions involving two products. In the simulations, *Q* is modeled as the binding energy of the valence electron to the nucleus, representing the absorbed kinetic energy so the ionization reaction is thus possible. While the minimum *Q*-value should be the lower limit of DOS distribution of liquid water (around 10 eV for 1b_1_ orbital), its maximum value necessary for sampling must be defined as total energy loss such that,

7 *Q* ≤ *E*_0_ − *E*′

Substitution from eqn (5) for *E*′ and rearrangement yields,8
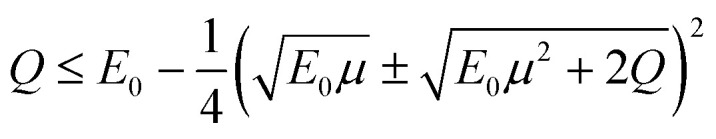
Since *µ* = −1 for the minimum *E*′, eqn (8) simplifies to,9
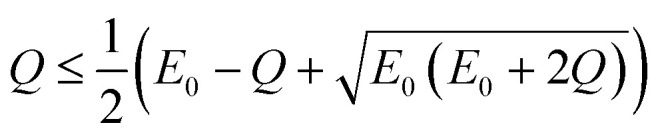


Rearranging eqn (9) yields the following.10
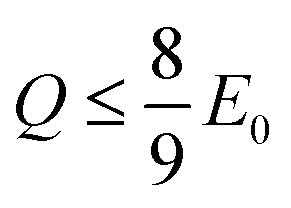


**Fig. 3:**
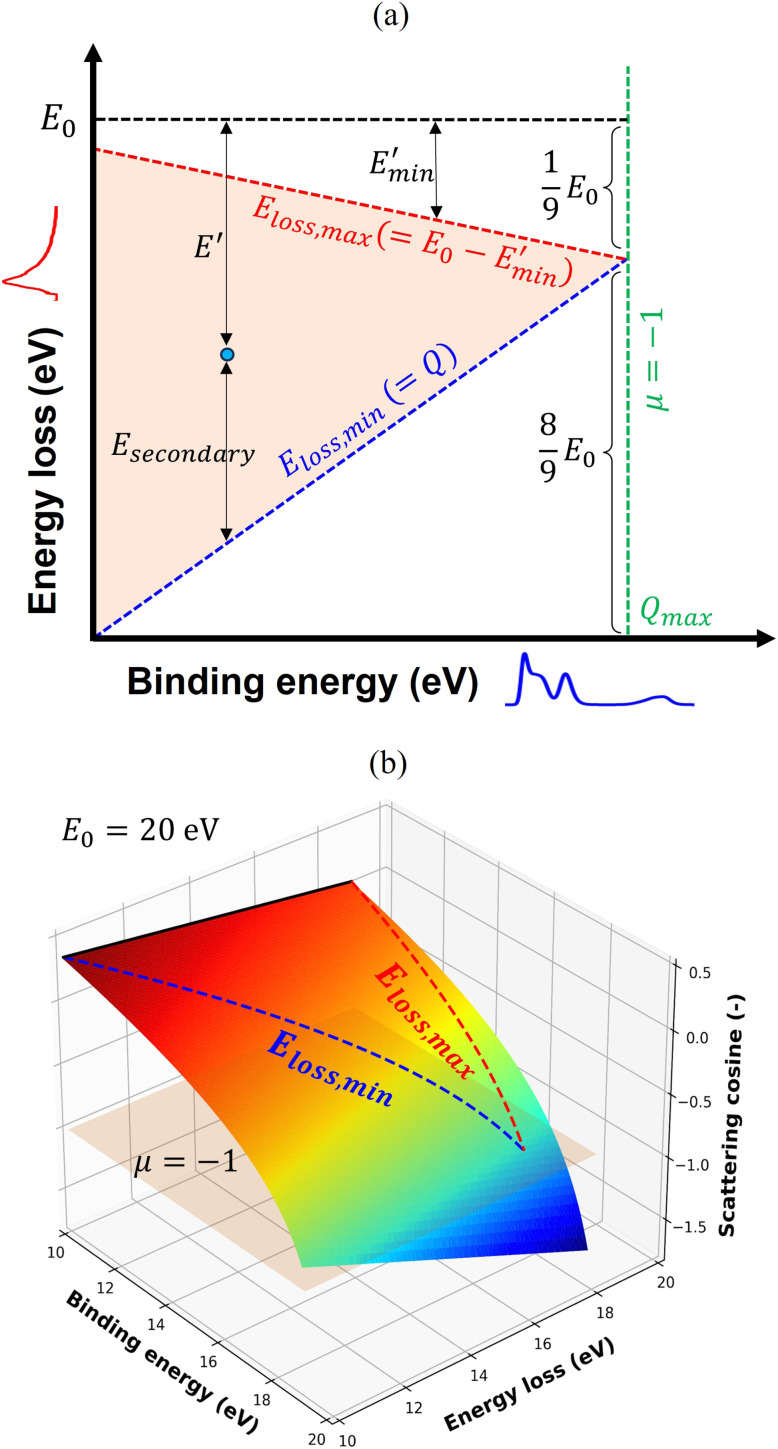
(a) Schematic representation of the sampling range for ionization energy loss, defined by the kinematic thresholds of *Q* and *µ*. (b) Surface plot of *µ* as a function of *Q* and ionization energy loss for *E*_0_ of 20 eV. The triangular sampling region is delineated on the surface by lines, while the regions beyond the sampling limit are included to illustrate the full topology. The color scale represents the *µ* value, and the horizontal plane indicates *µ* = −1.

Using eqn (10) as the kinematic threshold, the total energy loss sampling range can be determined, as shown in Fig. 3 (a). Notably, the maximum possible energy loss decreases with increasing *Q*, ensuring that *µ* in eqn (6) remains within the range of −1 or greater and that *E*′ in eqn (7) reaches a minimum of 1/9*E*_0_. Fig. 3 (b) illustrates the sampling range of ionization energy loss and *Q*, which are sampled from the ELF, shown in Fig. 2 (b), and DOS data, respectively, on the scattering cosine surface.

#### 2.2.3 Ionization (100 eV and below)

For incident electron energies below 100 eV, MC and dielectric-response-based models often require a q-dependent ELF to properly account for the increasing importance of momentum transfer, in which case the OELF is extended to finite-q regions. In this work, the extended ELF for LEEs is adopted from a recent report^69^ that developed an improved random phase approximation (RPA)-ELF incorporating local-field effect (LFE) and realistic liquid structures. The model provides a more accurate description of energy loss spectra and IMFP for LEEs in liquid water (H. Nguyen-Truong, personal communication, May 3, 2025). Starting with this improved RPA-ELF, we extended the *q* range, using an exponential decay function to ensure continuity, covering *q* values up to 6.0 Bohr^−1^, which corresponds to the maximum *q* of a 100 eV electron. The SDCS was then obtained by integrating over the possible momentum transfer range 
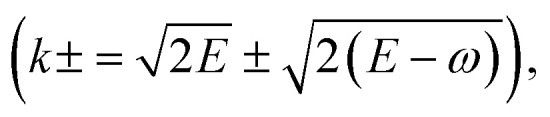
 from which the electronic energy loss was subsequently sampled.

It is important to note that the modified Born–Ochkur factor, *f*_ex_(*q*,*E*), shown in eqn (11),11
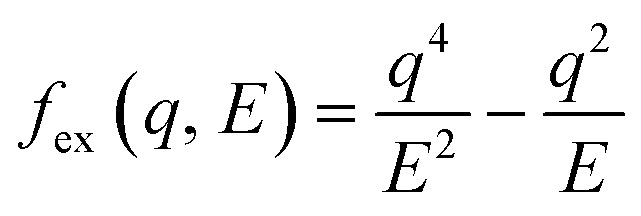
was applied within the dielectric formalism to approximately account for the exchange effect, interference, and momentum-transfer dispersion observed in LEE scattering but not fully captured by the available cross section data. Several empirical correction strategies—including, in some cases, the complete omission of the correction—have also been adopted in recent studies,^68,69,97^ including our previous work,^31^ supporting the practical validity of such approaches. The correction used in our earlier study was effective for the two low energy cases examined (14.3 and 19 eV), yielding good agreement with the experimental Pt–ASW EELS spectra. However, when this correction is extended to higher *E*_0_ up to 100 eV, the resulting SDCS exhibits a noticeably different spectral shape compared to the OELF used for high energy electron cases (shown in Fig. 2 (b)), including a prominent secondary peak near 30 eV. Because the calculation of electron transport properties is intended to reflect intrinsic electron kinematics, independent of non-intrinsic effects such as surface reflection, secondary electron emission, and substrate transmission that were considered in the EELS modeling, maintaining a physically consistent description of energy loss across the entire energy range becomes important. Accordingly, to ensure physical continuity in the energy loss behavior across the 100 eV boundary, we adopted the alternative correction factor used in ref. 68 and 97 (see Fig. S4 in the SI).

**Fig. 4:**
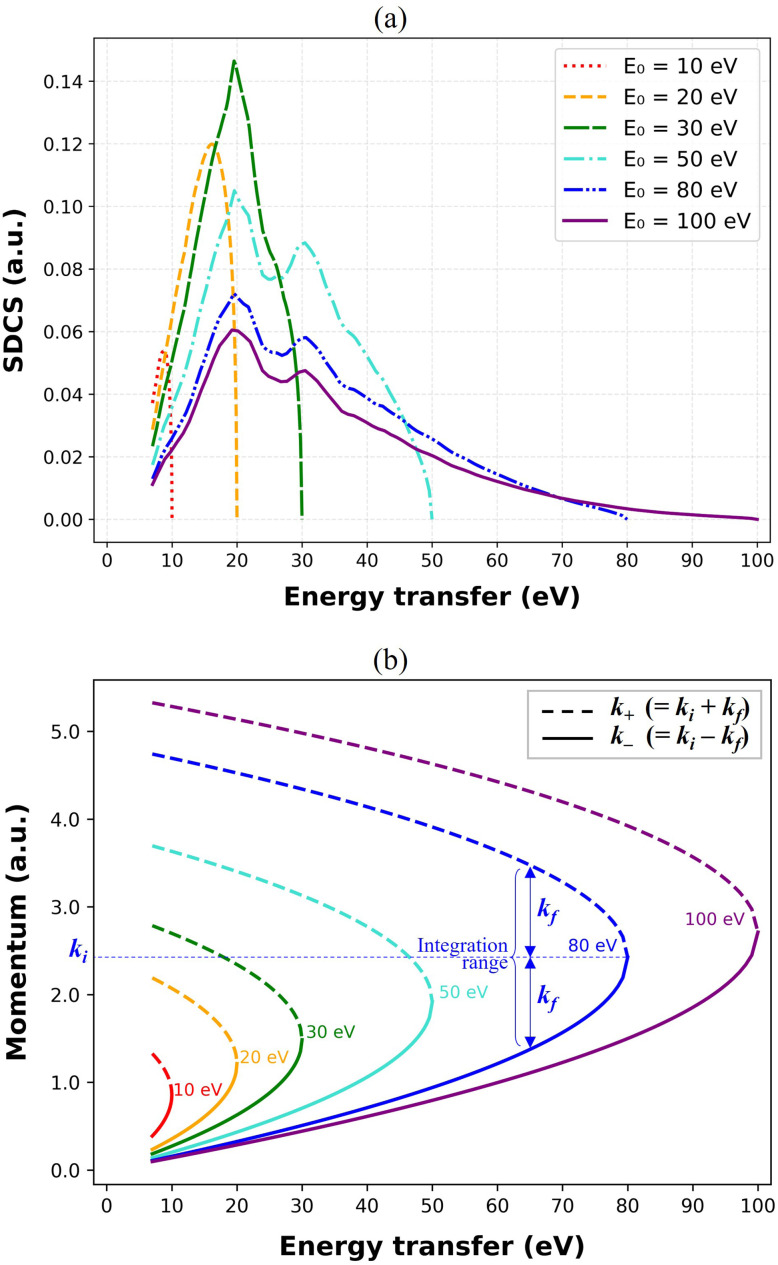
(a) SDCS as a function of energy transfer for *E*_0_ of 10, 20, 30, 50, 80, and 100 eV. The results were obtained using the modified Born–Ochkur correction scheme applied to the RPA-ELF method.^69^ (b) Corresponding momentum transfer limits (*k*_−_ and *k*_+_) as a function of energy transfer, which define the integration ranges used in the SDCS calculation for each *E*_0_. Solid and dashed lines represent the lower and upper limits of momentum transfer, respectively. Initial and final wave vectors, *k*_*i*_ and *k*_*f*_, are shown for the 80 eV case for clarity.

It should also be emphasized that, in MC track-structure simulations, energy loss and transport quantities are primarily determined by the mean value sampled per event—namely, the mean energy loss and 〈*R*_th_〉, the latter of which depends solely on the underlying cross sections. Since the cross sections remain unchanged across all correction schemes, the only relevant factor is the difference in mean energy loss. At *E*_0_ = 100 eV, the SDCS mean energy loss obtained using the Born–Ochkur correction adopted in this study is very similar to that of the OELF (33.4 *vs.* 32.4 eV), resulting in no discontinuity in 〈*R*_th_〉 at 100 eV, as shown later in Fig. 6. Fig. 4 (a and b) shows the SDCS derived in this work and the corresponding momentum transfer limits used in the integration. A detailed comparison of the SDCS dependence on *E*_0_, correction choice, and resulting distribution shapes is provided in the SI (see Fig. S5).

One important consideration in modeling electronic energy loss is to separately account for the contributions of electron-impact excitation and ionization. Given that no experimental measurements have yet reported these two distinct process cross sections of liquid water as independent quantities—and that the optical responses of both processes are indistinguishable in the DOSD or ELF—such separation is not straightforward. In an ionization process, if the deposited energy exceeds the ionization potential, the ionization efficiency may, in principle, be assumed to approach unity. In MC track-structure simulations, however, the energy loss per interaction is not a modeling parameter but a sampled outcome. Therefore, the event type must be determined prior to sampling the deposited energy, based on the ionization efficiency corresponding to the given *E*_0_. For ionization events, *Q* and the total energy loss are then computed by applying the appropriate kinematic constraints (see eqn (5) and (6)) and enforcing energy–momentum conservation.

**Fig. 5:**
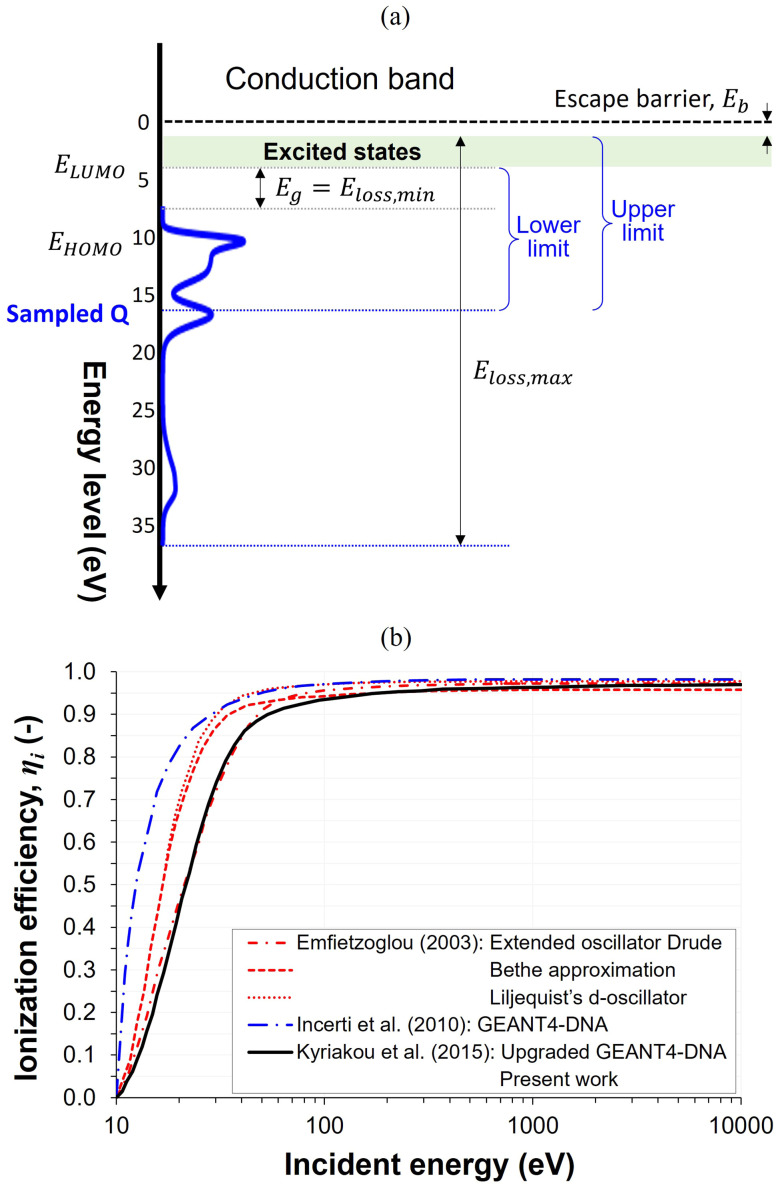
(a) Schematic representation of the sampling range for excitation energy loss for electrons with *E*_0_ of 100 eV or higher. (b) Energy dependence of ionization efficiency. Results from^99^ (dashed, dotted, and dash-dotted red) obtained using different dispersion algorithms applied to the same optical ELF. Results from^98^ (solid black) and^100^ (dash-dotted blue) are based on an existing GEANT4-DNA physics model, where^98^ has adopted a Drude optical model^99^ (dash-dotted red) to upgrade the existing framework, and is the model adopted in the present work for the > 100 eV region.

It is important to recognize that, at any *E*_0_ above the ionization threshold, there remains a finite probability of electronic excitation in the 6–13 eV range, where ionization cannot occur. As a consequence, the *E*_0_-dependent likelihood of ionization can never reach unity (as shown later in Fig. 5(b)). Recent theoretical studies have further shown that retaining a small fraction of excitation events (typically within 5%) even for *E*_0_ above 100 eV yields significantly improved agreement with International Commission on Radiation Units and Measurements (ICRU)-recommended inelastic cross section data sets.^98–100^ This behavior is consistent with the fact that, in the high energy (“white-light”) limit, the excitation–ionization partition is ultimately governed by the underlying oscillator strength distribution. Accordingly, in the present work, the type of electronic events was subsequently modeled as two mutually exclusive channels, specified through an input branching ratio determined by the energy-dependent fractional contributions of ionization and excitation to the total inelastic cross section (*i.e.*, the ionization efficiency), as reported in 98.

#### 2.2.4 Electronic excitation

Even after identifying the event type, determining the range of corresponding excitation energy loss remains challenging, owing to the theoretically infinite number of possible excited configurations in a water molecule and the extremely small momentum transfer in such events, which negligibly affects the center-of-mass motion of the excited water molecule. Consequently, most previous studies have focused on specific electronic states, typically those corresponding to excitation energies of 7–12 eV for singlet states. In contrast, as illustrated in Fig. 5 (a), the excitation energy loss in this study was sampled directly from the DOS distribution, taking the band gap energy, *E*_g_, (7.4 eV) and the highest occupied molecular orbital energy, *E*_HOMO_, (11.2 eV) into account to encompass all possible energy-transfer channels associated with electronic excitation. A similar approach was previously suggested in ref. 68. The chosen lower bound of 7.4 eV is close to the commonly adopted excitation threshold reported in numerous theoretical studies and is also widely used as the subexcitation energy limit in many computational models. Although real excitation thresholds are broadened, often represented by a Gaussian distribution, and thus allow a small but finite probability of excitation down to ∼6 eV, 7.4 eV remains a practically established convention for defining the onset of electronic excitations in MC track-structure simulations.

For electrons with energies below 100 eV, modeling electronic excitation energy loss becomes even more challenging because the fractional contributions of ionization and excitation to the total inelastic cross section show considerable variation between previous studies. As illustrated in Fig. 5 (b), for instance, the ionization efficiency at *E*_0_ of 20 eV ranges widely from approximately 0.4 to 0.8. Selecting an appropriate value is critical, as it can substantially affect the calculated energy loss and transport properties of LEEs. It is worth noting that the study from which we adopted the ionization efficiency for the high energy region^98^ refined an existing GEANT4-DNA model^100^ by implementing a truncation algorithm that rendered the excitation and ionization cross sections comparable to those obtained in an earlier work employing one of the three major dielectric-model formalisms.^99^ Nevertheless, the resulting ionization efficiencies reported in ref. 98 remain significantly lower than those in previous studies.^99,100^ Accordingly, in the present work, for LEEs with energies below 100 eV, we did not rely on an ionization efficiency parameter. Instead, the energy loss sampled from the SDCS was classified as ionization energy loss when the sampled value exceeded the ionization potential of 11.16 eV, and as excitation otherwise. This value (11.16 eV) corresponds to the lowest reported binding energy of liquid water^101^ and has been confirmed through Pt–ASW EELS simulations performed in our previous study.^31^ An event-by-event classification based on a fixed energy threshold has also been adopted in earlier studies of electron transport.^68,99^

### 2.3. Angular deflection

The scattering angle governs the post-collision trajectory of electrons and is therefore a critical modeling parameter for predicting electron transport properties, particularly 〈*R*_th_〉. Similar to the cross sections discussed earlier, there is no clear consensus on the angular deflection characteristics for electron scattering in liquid-phase water, as existing interpretations vary due to discrepancies or lack of reliable experimental and computational ADCS data. For vibrationally inelastic scattering, the anisotropy factor reported for ASW by Sanche and coworkers^21^ was adopted to ensure consistency with the corresponding cross section data sets, thereby allowing *µ* to remain energy dependent. An earlier study^72^ approximated this process using a delta wave function, effectively a delta-function angular distribution corresponding to forward scattering, while other works^47,68,89^ equivalently treated such events as producing no change in the particle trajectory because of their negligible momentum transfer. However, all these assumptions yield virtually identical transport outcomes, since the reported anisotropy factor for the inferior inelastic components adopted in this study^21^ increases rapidly with electron energy and approaches unity—indicating forward scattering—above only a few tenths of eV.

In the case of electronic excitations, the primary electrons are assumed to undergo no angular deflection from their initial trajectories (*i.e.*, glancing collisions),^68,72,102^ and the same approach is adopted in this study. This assumption arises primarily from the lack of experimental data and physical interpretation regarding the energy dependence and angular distribution of electronic excitation processes in liquid-phase water, apart from gas-phase observations.^12,13^ It should be noted, however, that excitation dominates over ionization only within a narrow *E*_0_ window (approximately 7–20 eV, depending on ionization efficiency).

For ionization events, scattering is inherently anisotropic because of energy and momentum conservation, although the extent of deviation varies among studies. While some have limited the scattering angle to the range 0 to π/4,^68^ our treatment is based solely on energy–momentum balance for *E*_0_ below 100 eV. For *E*_0_ above 100 eV, the angular deflection is determined directly from scattering kinematics (see eqn (5) and (6)), accounting for both *E*_0_ and the maximum permissible energy loss, *E*_loss,max_, (see Fig. S3(a and b) in the SI for the corresponding distribution).

In our simulations, elastic collisions are also treated as anisotropic, with the *µ* sampled from the ADCS data.^68^ The azimuthal angle, *φ*, is sampled assuming spherical and cylindrical symmetry over the range considered. As noted earlier, the combination of no energy transfer and anisotropic scattering renders elastic collisions uniquely influential in determining transport characteristics compared to other collision types. Under these angular deflection assumptions, the post-collision direction (*u*′, *v*′, *w*′), transformed from the pre-collision direction (*u*, *v*, *w*), is defined as^103^12
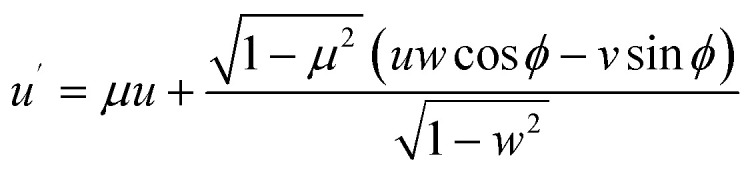
13
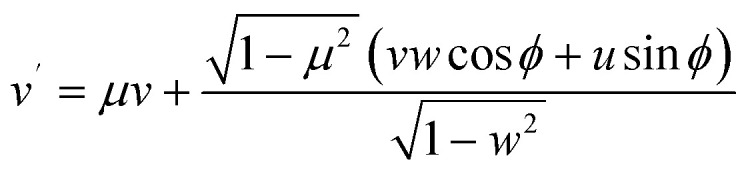
14
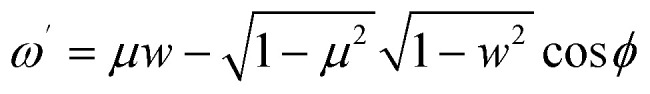


## 3. Results and discussion

### 3.1. Transport properties

In studies of electron transport in condensed matter, the most commonly reported quantity is the MFP. However, from the perspective of chemical physics, this number alone carries limited physical significance. In radiation chemistry modeling, what is instead required is knowledge of quantities of critical importance—namely, the energy lost in each inelastic event and the average total distance traveled by an electron with a given *E*_0_. To gain physical insight into, and to achieve accurate predictions of, these quantities, it is necessary to derive, from the MFP and related data, the partial scattering cross sections, and to determine the energy spectrum of secondary electrons generated in ionization events for a given *E*_0_, the relative yields of electronically excited states of various energies, and the angular distribution of scattering. Only with such information can we obtain insight into the actual outcomes of scattering processes, and only then can transport quantities such as the MFP be meaningfully interpreted and effectively used.

It is important to clarify the definitions of other terms used to describe electron transport properties, particularly those related to *R*_th_, as inconsistent usage appears across the literature, sometimes even within studies by the same authors. In ref. 35 and 72, this quantity is described using the terms “penetration” and “depth,” which are treated as synonymous. In those studies, the term “range” is used to denote the total cumulative path length of the electron trajectory. However, to our knowledge, this usage is not consistent with earlier conventions in the literature. In one of the pioneering MC studies of electron scattering,^41^ the term “penetration” is used exclusively to describe directional components of the displacement (*i.e.*, axial and radial penetration), whereas “range” refers to the vector displacement between the initial and final electron positions, corresponding to *R*_th_ as defined in the present work. To avoid ambiguity, the terms “penetration” and “range” are therefore not used in this study to describe distance.

Care should also be taken when describing the size of a spur. A spur is a localized cluster of radiolytic species formed along an electron track. However, no method has been proposed to directly measure its size.^73^ Consequently, in stochastic models, its spatial extent is commonly characterized by an isotropic 3-D Gaussian distribution describing the initial locations of radiolytic species. The standard deviation of this distribution, *σ*, is historically referred to as the “spur width.” Values of *σ*(e_aq_^−^) in the range 2.0–3.0 nm were reported in early deterministic analyses of scavenger experiments, and later revised upward to 4.0–5.2 nm (see Fig. S8 in the SI and references therein). However, strictly speaking, *σ* is not a physical distance representing the spur size, but rather a width parameter describing the spatial spread of radiolytic species within a spur; direct comparison with *R*_th_ is therefore inappropriate. A more meaningful comparison can be made using characteristic distances derived from the Gaussian distribution, such as the root-mean-square (RMS) radius, 
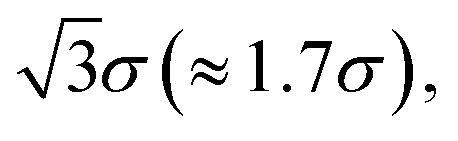
 reported to be 6.9–8.3 nm,^52,53,104^ or the mean radius, 
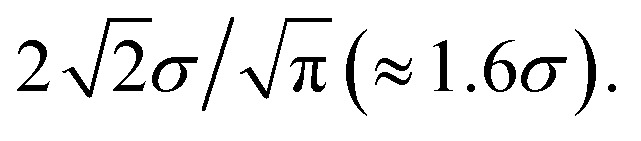
^39,71^ These quantities are often referred to as the “spur radius,” reflecting historical models that treated spurs as uniform spheres. Even after the transition to Gaussian descriptions, the term “radius” has been retained by convention and is frequently used interchangeably with *σ*, since *σ* uniquely defines the characteristic length scale of the distribution and thereby determines all other commonly used size descriptors.

#### 3.1.1 Geminate separation distance

In the simulations, the model repeatedly samples traveling distances at each step and propagates the electron accordingly, thereby determining the spatial location at which its kinetic energy has dropped below the thermalization cutoff of 0.2 eV. This final position is then used to calculate *R*_th_, defined as the straight-line distance from the initial position at the ionization core. For the evaluation of the mean effective geminate separation distance, denoted as 〈*R*_gem_〉 in this study, the ejection length associated with EAD and autoionization processes is taken into account, as discussed in a later section.

**Fig. 6:**
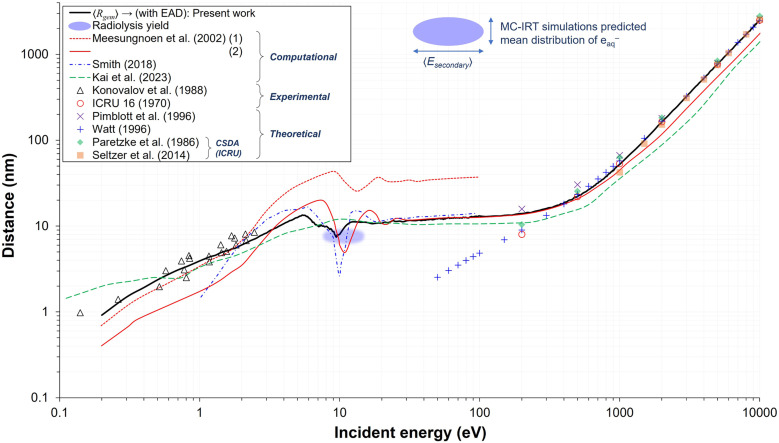
Various electron transport properties reported as a *E*_0_-dependent distances over the range of 0.2 eV to 10 keV. The present results, representing the mean geminate separation distance, 〈*R*_gem_〉, including the TNA-EAD process, are compared with reported 〈*R*_gem_〉 values from computational studies,^35,71^ the mean thermalization distance 〈*R*_th_〉,^47^ and theoretical approximations,^41,59^ including CSDA values based on the recommended stopping-power compilations of ICRU Reports 16 and 37 (ref. 60) and the updated ICRU Report 90.^61^ Experimental benchmarks include photoinjection measurements representing 〈*R*_th_〉 in liquid water,^57^ as well as historical low energy 〈*R*_th_〉 measurements in moist air and thin collodion films,^58^ which were cited in ICRU Report 16 for validation purposes. The shaded region indicates the representative energy range (approximately 8.0–14.1 eV; see Fig. S7 (a) in the SI) and the corresponding 〈*R*_gem_〉 values (6.9–8.3 nm; see Fig. S7 (b) and S8 in the SI) associated with the radiolysis yield match. For comparison of the present results with OpenTOPAS using a matched cutoff energy of 7 eV, see Fig. S6 in the SI.

In the very low energy range, specifically below 2.5 eV, photoinjection experiments have been performed.^57,86^ These data, even after nearly four decades, continue to serve as an important experimental benchmark, as they remain the only direct measurements of *R*_th_ for electrons in aqueous solution. In the high energy region above several hundred eV, where energy loss fluctuations can be neglected, CSDA values provide reasonable approximations of *R*_th_, as they can be derived theoretically by integrating the reciprocal of the total stopping power. As shown in Fig. 6, the present results are in good agreement with both photoinjection measurements in the lower energy region and with most theoretical approximations at higher energies.

Accurately predicting 〈*R*_gem_〉 in the intermediate energy range (2.5–100 eV) remains challenging due to the lack of benchmark data for direct comparison. Moreover, the validity of the conventional scattering cross section concept and even the trajectory-based approach itself may break down at low energies because of the non-classical characteristics of LEEs, such as strong exchange, interference, and coherent-scattering effects,^74,97^ together with the potential for diffusive motion accompanied by significant angular deflection near the end of their trajectories. Even among previously reported simulation studies, this energy regime is subject to considerable debate, not only regarding the absolute value of distance but also its dependence on *E*_0_. Some studies, including the present work, report a relative plateau in 〈*R*_gem_〉 (or 〈*R*_th_〉) and electron effective attenuation length (EAL)^37,47,105^ from 10 eV up to a few hundred eV, whereas others show pronounced fluctuations with increasing *E*_0_.^33–35,71,106^

Starting from the low energy limit, 〈*R*_gem_〉 increases, reaches a maximum near 5–6 eV, and then exhibits a pronounced drop throughout the 6–10 eV region. This sharp decrease arises directly from TNA-driven track termination events. In the 10–13 eV range, 〈*R*_gem_〉 begins to increase again because the contribution of excitations that do not alter the particle direction remains higher than that of ionization within this narrow range. However, the increase ceases and forms a plateau by around 100 eV due to the onset of ionization. Notably, the mean energy of secondary electrons lies within this fluctuating regime containing a local minimum near 10 eV (see Fig. S7 in the SI). This placement underscores the intrinsic difficulty in accurately predicting 〈*R*_gem_〉 *via* MC track-structure simulations, where the full complexity of elastic scattering, TNA-driven processes, and the competing electronic channels, each contributing in different proportions, collectively introduce substantial uncertainty.

Given these uncertainties, including the previously discussed differences between gas-phase and liquid-phase DEA cross sections and the empirical assumptions required to determine the fractional contribution of electronic events, the best approach—other than performing a comparative analysis while recognizing that differences in modeling assumptions may exist—is to confirm the agreement between the present model results and reported mean e_aq_^−^ spatial distributions representing spur radii (blue ellipse),^52,53^ which are required in IRT simulations to model the picosecond radiolysis kinetics and scavenging yields. The horizontal axis range of 8.0–14.1 eV represents the mean energy of secondary electrons reported in different studies (see Fig. S7 in the SI). As shown, the present model results overlap part of this region. We expect that relatively minor tweaks of the present model will prove fully consistent with radiolysis data in future work. It should be noted that some earlier studies, such as those by Jay-Gerin and coworkers^71^ (solid red) and Pimblott and coworkers^35^ (dash-dotted blue), also achieved a certain level of agreement within a limited energy range, but relied on less physically justified assumptions—namely, scaling ASW cross section data sets by a factor of two,^39,71,107^ or introducing an additional charge trapping cross section^34,35,108^ whose magnitude is itself relatively large, applied not only near 10 eV, but across the entire energy range below 11 eV as an intensive energy loss channel (see Fig. S8 in the SI).

#### 3.1.2 Sensitivity analyses

**Fig. 7:**
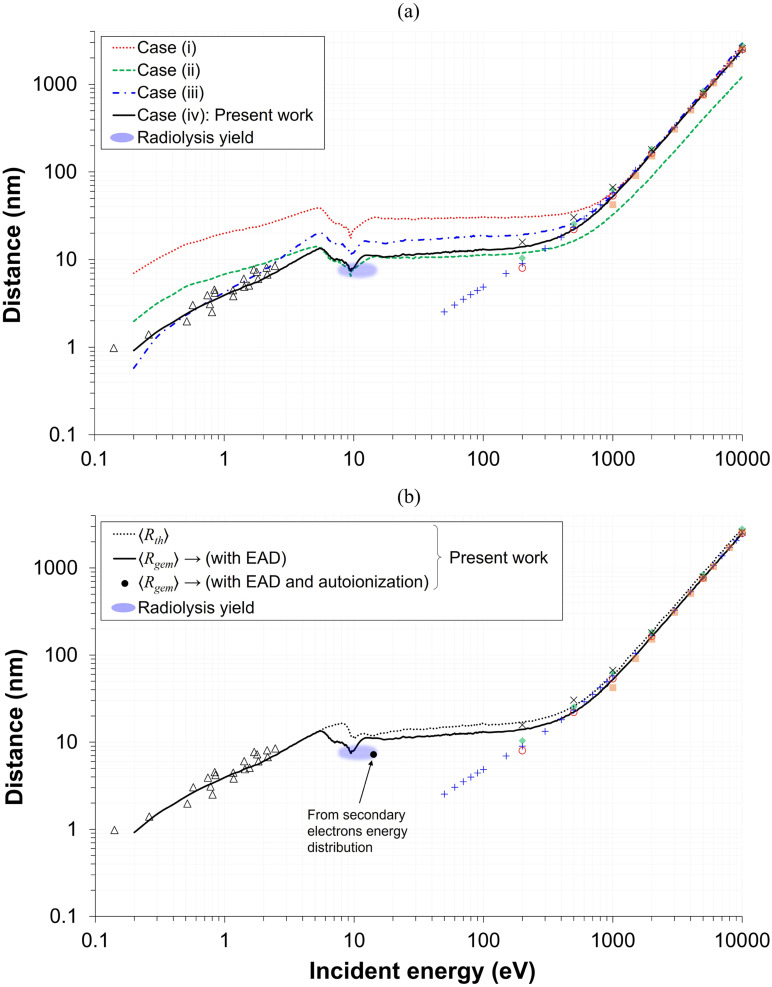
(a) Effect of elastic cross section on 〈*R*_gem_〉, illustrating how different choices of data sets and angular deflection assumptions modify electron transport properties. Four cases are compared: (i) ASW cross sections^21^ with isotropic deflection; (ii) ASW cross sections scaled (×20) with isotropic deflection; (iii) liquid cross section derived from MTMFP data^68^ with isotropic deflection; (iv) liquid cross section derived from EMFP data^68^ with anisotropic deflection, which are adopted in the present work. (b) Effect of TNA-driven EAD on 〈*R*_gem_〉, showing that inclusion of TNA pathway (solid black), together with electronic channels, produces a pronounced minimum between 5–10 eV that overlaps with the radiolysis yield. Also shown are 〈*R*_gem_〉 data point (black circle) calculated using *E*_0_ values sampled directly from the secondary electron energy distribution obtained with the MC model developed in this work, with autoionization processes originating from neutral excited molecules explicitly taken into account (see Fig. S8 in the SI for details).

As emphasized earlier, elastic scattering is a critical process governing LEE transport properties because it dominates in the low energy region and involves no energy loss. Therefore, evaluating the impact of elastic cross sections on electron transport properties is essential for identifying the cross-section data sets required to reproduce experimental measurements in liquid-phase water. Fig. 7 (a) compares 〈*R*_gem_〉 predicted using data sets reported in earlier studies,^21,68^ including the ASW cross sections reported by Sanche and coworkers. It should be noted that the ASW data sets were derived by simplifying the angular deflection process into a one-dimensional framework, in which the anisotropic components of elastic scattering, dominated by forward-peaked small-angle events, were effectively treated within the straight-line approximation rather than explicitly resolved. Consequently, using these data as input for transport simulations inevitably leads to an overestimation of 〈*R*_gem_〉 (or 〈*R*_th_〉) at low energies,^34,36–39^ owing to the longer MFP implicitly assumed for elastic events.

When ASW elastic cross section data sets of Sanche and coworkers^21^ were used (Case (i), Fig. 7(a)), the results consistently overestimated the radiolysis yield data as expected, requiring a large scaling factor (×20) to reproduce the radiolysis yield in liquid water (Case (ii), Fig. 7(a)). However, even with this correction, the agreement remained poor at both low and high energies, suggesting that consideration of the anisotropic component of elastic scattering is essential. This behavior is reminiscent of earlier works^39,71^ in which all the ASW cross sections were *ad hoc* upscaled by a factor of two to represent liquid-phase and thereby reproduce the typical spur radius employed in deterministic spur models of liquid water radiolysis. With this 2× scaling, the model produced a reasonable value of 〈*R*_gem_〉 at energies greater than ∼30 eV, but only at the cost of sacrificing agreement with photoinjection data^57,86^ at very low energies (<2.5 eV) (see solid red line in Fig. 6). The underlying origin of such discrepancies between ASW and liquid water was traditionally attributed to differences in density and structural order.^69^ However, given the relatively small difference in density of ASW and water (on the order of 6%) and their nearly identical DOSD,^29^ this explanation is not sufficient. More recent findings further suggest that variations in O–H stretching bond characteristics may also play a contributing role, as Raman measurements show that cooling shifts the dominant O–H stretching mode from the higher-to the lower-frequency band, thereby reducing the electron-molecule collision probability and leading to a longer IMFP in ASW.^69^ However, this argument is also not satisfactory, as a recent study has shown that the IMFP derived from liquid microjet experiments is nearly identical to that of ASW,^68^ and because that study does not address its effect on the EMFP.

The comparison between Cases (i) and (iii) in Fig. 7 (a) further confirms that, even when only the isotropic components are considered, the liquid-phase (MTMFP-derived) cross sections of ref. 68 provide a substantially more realistic representation than those derived from the ASW experiments of Sanche and coworkers.^21^ In principle, elastic collisions are governed by the electrostatic potential of nuclear charges—that is, the Coulomb repulsion from the electron cloud and the attraction to positively charged nuclei—and phase transitions are therefore generally not expected to greatly affect the cross section.^41^ However, published results indicate that the differences can become significant at lower energies below 20 eV (see Fig. 1 (a)). At such low energies, elastic scattering may be influenced by the polarization properties of the medium.^74^ Even ref. 30, which emphasized the resemblance in scattering properties between ASW and liquid water due to comparable densities and the disordered nature of the hydrogen-bond network, reports a noticeable discrepancy in the EAL between ASW simulations and liquid measurements below 20 eV (see Fig. 1 of ref. 30 and references therein). This “definitely different behavior below 20 eV,” anticipated in ref. 68 where the MTMFP data set was originally reported, is clearly confirmed in the present study. This suggests the notion that ASW behaves merely as viscous water with negligible effect on electron scattering does not hold at such low energies. The exact origin of this difference remains unknown. It may not reflect an intrinsic phase effect (ASW *vs.* liquid) but instead arise from limitations of the one-dimensional two-stream approximation employed in the ASW analysis, in which the anisotropic component of elastic scattering is excluded.^17–19,21^ Alternatively, it may originate from the inherent characteristics of the EELS experiment itself, which only probes backscattered electrons; if this were the dominant factor, a liquid-water measurement performed under the same experimental configuration would be expected to yield a similar result. As another possibility, it “seems not unlikely”^74^ that the EMFP derived from Pt–ASW EELS^21^ may itself be overestimated, in which case a new measurement might very well produce different results.

Regardless of the underlying cause, it is evident that this issue cannot be resolved by approaches such as selectively scaling individual events (Case (ii), Fig. 7 (a)) or uniformly scaling the entire cross section dataset.^39,71,107^ Achieving kinematic accuracy instead requires elastic cross sections that account not only for momentum transfer due to large-angle (or isotropic) deflections contributing to beam attenuation, but also for the cumulative effects of small-angle (or anisotropic) deflections, rather than relying on an effective straight-line approximation. Consequently, the ASW elastic cross section, as reported by Sanche and coworkers,^21^ is not appropriate for determining 〈*R*_gem_〉 of LEEs below 100 eV, and particularly below 20 eV, even when substantial scaling is applied. Among the four cases examined in this work, the EMFP-derived liquid-phase dataset^68^ (Case (iv), Fig. 7 (a)) yields the most accurate results across the low, intermediate, and high energy ranges.

Fig. 7 (b) shows the difference between 〈*R*_th_〉 and 〈*R*_gem_〉, illustrating the effect of the TNA-driven track-termination processes on the effective electron transport distance. When TNA is omitted—or included without being treated as a track terminating event—the pronounced minimum observed between 5–15 eV becomes more shallow or nearly flat, consistent with the trends reported in ref. 47 and 72. In the absence of TNA, the remaining minimum appearing above ∼10 eV originates from the onset of electronic excitation channels, which also initiate near this energy. The results further indicate that TNA-driven mechanisms influence not only the low energy regime but also modify 〈*R*_th_〉 at higher energies to a certain extent. In this context, comparisons with theoretical approximations, particularly CSDA, require caution. CSDA is a standard measure for describing the transport of heavy charged particles^96^ and is strictly valid only for high energy electrons,^109^ as it neglects individual scattering events, angular deflections, energy loss fluctuations, and stopping power discrepancies that are significant for LEEs.^35,41^ For this reason, comparisons with reported CSDA values are restricted here to electron energies above 100 eV. Moreover, given the definition of the CSDA, it is more appropriate to compare these values with 〈*R*_th_〉 (dotted line). As shown in the figure, the overall agreement remains close in both cases, with slightly better agreement observed with values reported in ref. 59 and 61 for 〈*R*_gem_〉 and in ref. 41 and 60 for 〈*R*_th_〉, respectively.

In several earlier studies, it has been argued that the anisotropic component of elastic scattering can be neglected in LEE transport because forward-peaked collisions were believed to have little influence on the electron beam attenuation.^17–19,21,30^ This argument, however, also warrants closer examination. When scattering occurs exactly in the forward direction (*µ* = 1), the electron trajectory remains unchanged and thus makes no contribution to electron transport or beam attenuation. In practice, however, scattering rarely occurs strictly at 0° but instead proceeds through a sequence of small-angle deflections that cumulatively alter the electron's direction of motion. Recent computationally obtained elastic ADCS data^68^ indicate that forward-scattering deflections can reach 20–40°, even at energies below 10 eV. Therefore, the statement that forward-peaked scattering is irrelevant to electron transport should not be interpreted as implying zero contribution, but rather that its effect is relatively minor and strongly energy dependent.

#### 3.1.3 Effect of ejected electrons

**Fig. 8:**
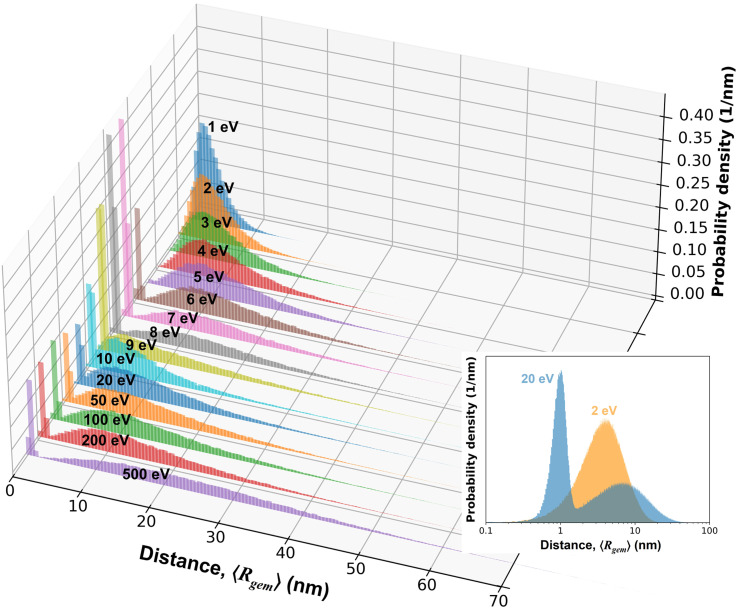
Distributions of the geminate separation distance, *R*_gem_, for electrons with *E*_0_ ranging from 1 to 500 eV. The labels indicate the values of *E*_0_, emphasizing a local maximum in 〈*R*_gem_〉 near 5–6 eV and a local minimum near 10 eV. For *E*_0_ ≥ 6 eV, electrons ejected from TNA states of water *via* EAD produce a pronounced low distance peak, reflecting the characteristics of ejection length. This feature is absent for *E*_0_ ≤ 5 eV, where TNA resonance is not available. Inset: Distributions of *R*_gem_ for the 2 and 20 eV cases are shown on a logarithmic scale to contrast the uni- and bi-modal shapes.

Another important factor considered in the present work when calculating mean distribution for e_aq_^−^ is the assumption that e_aq_^−^ originating from TNA-driven EAD process does not lose their contact with their sibling reactant. That is, e_aq_^−^ generated through different processes have their own distinct separation distances from their geminate partners, thereby contributing differently to the recombination yield of radiolytic species. Consequently, for electrons with *E*_0_ above the TNA threshold, the distribution of 〈*R*_gem_〉 would exhibit a bimodal shape, where two different separation distances are superimposed as illustrated in Fig. 8. As previously described, we assume unstable TNA resonances exist at energies around 5–15 eV, where electron energy is lost to electronic excitation. In this framework, the excited water molecule is assumed to undergo rapid dissociation, producing reactive radical fragments, with which the ejected electron produced through EAD can subsequently recombine in a local manner. The relevant distance in this case—referred to as the ejection length or localization distance, defined as the separation between a TNA or excited water molecule and the spatial localization of the ejected electron—has typically been assumed to be around 0.7–1.2 nm, depending on excitation energy and temperature.^22,47,110–112^ This suggests that the spatial extent of the spur estimated without considering this factor may be overestimated. In the present study, the ejection length is sampled from a Gaussian distribution with a mean of 1.0 nm and a standard deviation of 0.2 nm.

In order to treat TNA-driven EAD processes in this manner, a central assumption adopted in the present work is that the TNA process serves to localize the electron wavefunction, such that the wavefunction acquires the character of a local molecular excited state or a localized exciton that lies outside the conduction band. The subsequent decay of this localized state involves excitation of another valence electron, while the initially captured electron becomes trapped at a local trap site, thereby terminating delocalized transport. At sufficiently low electron energies, this picture is unambiguous: no alternative pathway exists, and the process necessarily results in an electron with zero kinetic energy, corresponding to complete track termination. However, the treatment of EAD, particularly at deposited energies near or above the ionization potential, has not been clearly defined in the literature with respect to whether it should be regarded as a simple energy-loss mechanism or as a track-terminating event. In this context, recent first-principles simulations by Kai *et al.*^73^ report energy-dependent relocalization of ejected electrons, demonstrating that a substantial fraction of these electrons return to localized or quasi-bound states, especially at deposited energies below approximately 15 eV. This behavior is fully consistent with the physical interpretation adopted in the present work. Within this framework, the magnitude of the TNA cross section and the branching ratio between DEA and EAD pathways emerge as the dominant parameters controlling the relative contributions of terminating *versus* transport channels. Nevertheless, these quantities remain poorly constrained experimentally, and direct measurements of TNA-mediated branching are not currently available. Consequently, quantitative modeling in this energy regime necessarily relies on informed assumptions, which we constrain by requiring consistency with available experimental observables and by adopting parameter values that reproduce the measured spatial and energetic characteristics of LEEs.

Unlike the TNA-driven EAD process, in which removal of the attached electron from H_2_O*^−^ returns the species to its neutral form, autoionization from neutral electronically excited H_2_O* generates an additional secondary electron and produces a cation. The resulting electron does not reach the conduction band and is therefore not observed in time-resolved photoelectron measurements either; it may instead subsequently recombine with its geminate partner in the same way as those that have undergone EAD or thermalization. For consistency, and in the absence of experimental guidance, the ejection length in this case is assumed to follow the same Gaussian distribution used for EAD process; however, the relative probabilities of autoionization, including competing decay pathways, dissociative and non-dissociative, remain uncertain. Some computational studies^39,42^ have adopted a simplified assumption in which 50% of the excited B^1^A_1_ state undergoes autoionization, motivated by the reported photoionization efficiency at an energy 1.3 eV above the first ionization potential (∼13.9 eV), where this state is located.^113^ However, this approximation neglects the presence of multiple superexcited states that exist throughout the higher-energy region of water, extending up to ∼35 eV,^113^ any of which may also contribute to autoionization with unknown probabilities. Therefore, in the present study, the relative likelihood of each pathway—e_aq_^−^ formation from H_2_O*^−^ (TNA-driven EAD) or from neutral excited water molecule, H_2_O*—is determined using a comprehensive set of reported data. In particular, the photoionization efficiency for the neutral excited channel is taken from ref. 113 based on (e, 2e) coincidence energy loss measurements of H_2_O. For the TNA pathway, the branching ratio between DEA and EAD is taken from Refs. 85 and 114.

### 3.2. *G*-Value of pre-solvated electron, e_aq_^−^

**Fig. 9:**
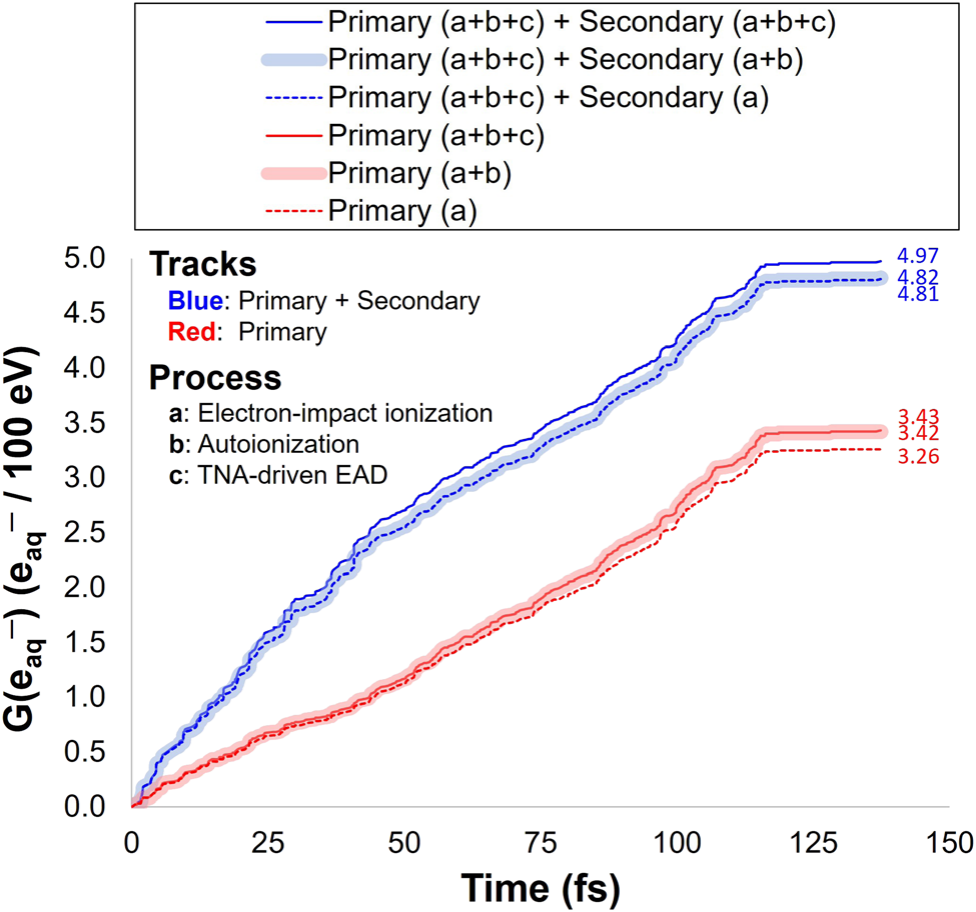
Temporal evolution of the cumulative *G*-value by track and process simulated with a 10 keV electron. The contributions of primary and secondary tracks to the *G*-value are approximately 7 : 3 (*i.e.*, 3.43 : 1.54). The figure shows a representative case; the final *G*-value of 4.9 is obtained as the average over 10^3^ samples.

To accurately predict the yield of e_aq_^−^, it is necessary to account for a certain level of early-time recombination of pre-solvated electrons occurring before the conventional ‘time zero’ for the chemical diffusion timescale; otherwise, model predictions tend to overestimate the yield, effectively representing only the upper limit of *G*(e_aq_^−^). Fig. 9 shows the temporal rise of the initial *G*-value, accounting for secondary electrons produced from both electron-impact excitation and the EAD process in primary and secondary tracks. As shown, the appearance of e_aq_^−^ is not instantaneous but develops over a sub-picosecond timescale. This gradual emergence has been confirmed experimentally through femtosecond pulse-radiolysis measurements.^115^ Our model reproduces the same solvation delay: the initial *G*-value increases steadily and saturates within the sub-picosecond timescale, in full agreement with the experimental observation. It is further noted that TNA-driven EAD events become relevant only along secondary electron tracks, increasing the *G*-value from 4.82 to 4.97 per 100 eV in the representative case shown in the figure. Along the primary track of a 10 keV electron, the likelihood of EAD events reaches only ∼0.1 (see Fig. S2 in the SI), and therefore the contribution of EAD to *G*(e_aq_^−^) is negligible (∼0.001). In contrast, along secondary tracks, where the mean electron energy is 14.1 eV (see Fig. S7 in the SI), EAD can still occur with a frequency exceeding 0.1 and thus contribute to the *G*-values, while the probability of both electron-impact excitation and the associated autoionization becomes negligible below 10 eV.

Widely accepted initial e_aq_^−^*G*-values (molecules per 100 eV) at approximately one picosecond in water radiolysis typically fall within the range of 4.0–5.0.^23,24,27,47,75,76,116–121^ The recombination yield of pre-solvated electron and its geminate partner can be estimated from a picosecond pulse-radiolysis study employing the silver-ion reduction technique,^75^ which reported *G*-values of 4.2 in pure water and 4.5 in concentrated Ag^+^ solution, corresponding to a recombination fraction of about 6.7% (*i.e.*, 0.3/4.5). Our modeled *G*-value of 4.9 is slightly elevated yet remains well within the range of previously reported values. It should be emphasized, however, that several modeling parameters—including the assumed photoionization efficiency, the branching ratio between DEA and EAD, and the relative contributions of ionization *versus* excitation—jointly influence the computed value and can be tweaked to reach a lower result.

Another noteworthy point is that MC track-structure calculations, including the present study as well as PARTRAC, GEANT4-DNA, and related models, tend to yield relatively high *G*-values, in the range of 4.83–4.9.^117–119^ By contrast, computational studies reporting lower values (4.05–4.15) have typically employed markedly different assumptions, such as setting the cutoff energy as high as 25 eV^120^ or determining the excitation-to-ionization partitioning based on a geometric criterion (*e.g.*, a reaction radius of 1 nm) rather than on the actual energy-loss distribution.^47^ Meanwhile, picosecond pulse-radiolysis experiments consistently report *G*-values in the low-to-mid 4 range (4.0–4.6),^23,24,27,75,76^ suggesting that this range likely reflects the physically relevant *G*(e_aq_^−^) at early times. In contrast, many computational approaches appear to systematically overestimate *G*-values, indicating that current models do not yet fully capture the underlying physics governing early-time electron production and loss. Theoretical estimates also exhibit substantial variation across studies, covering the 4–5 range.^116,121^ Given these variations and uncertainties, including the dependence on *E*_0_ and the employed electronic cross sections, careful evaluation is required when interpreting predicted *G*-values. We expect that progress in this area will require combining high-level *ab initio* calculations of TNA-driven dissociation channels with model-constrained analyses and validation against scavenger experiments to estimate the associated H^−^ and O^−^ yields and their contribution to presolvation H_2_ formation. Such efforts would help reduce the variability in early-time *G*(e_aq_^−^) estimates.

### 3.3. Track lifetime

Understanding when the track is terminated and the spatial extent of the spur is established provides deeper insight into electron transport characteristics, because it reveals temporal information that cannot be inferred from 〈*R*_gem_〉 (or 〈*R*_th_〉) alone. In particular, the track lifetime distributions confirm, on a temporal scale, the frequency of TNA-driven track-termination events, shown in Fig. 8 and S2 in the SI, identify the energy regime in which the increase in travel distance is offset by the electron velocity, and allow the track lifetimes inferred from *G*-value calculations to be independently validated.

**Fig. 10:**
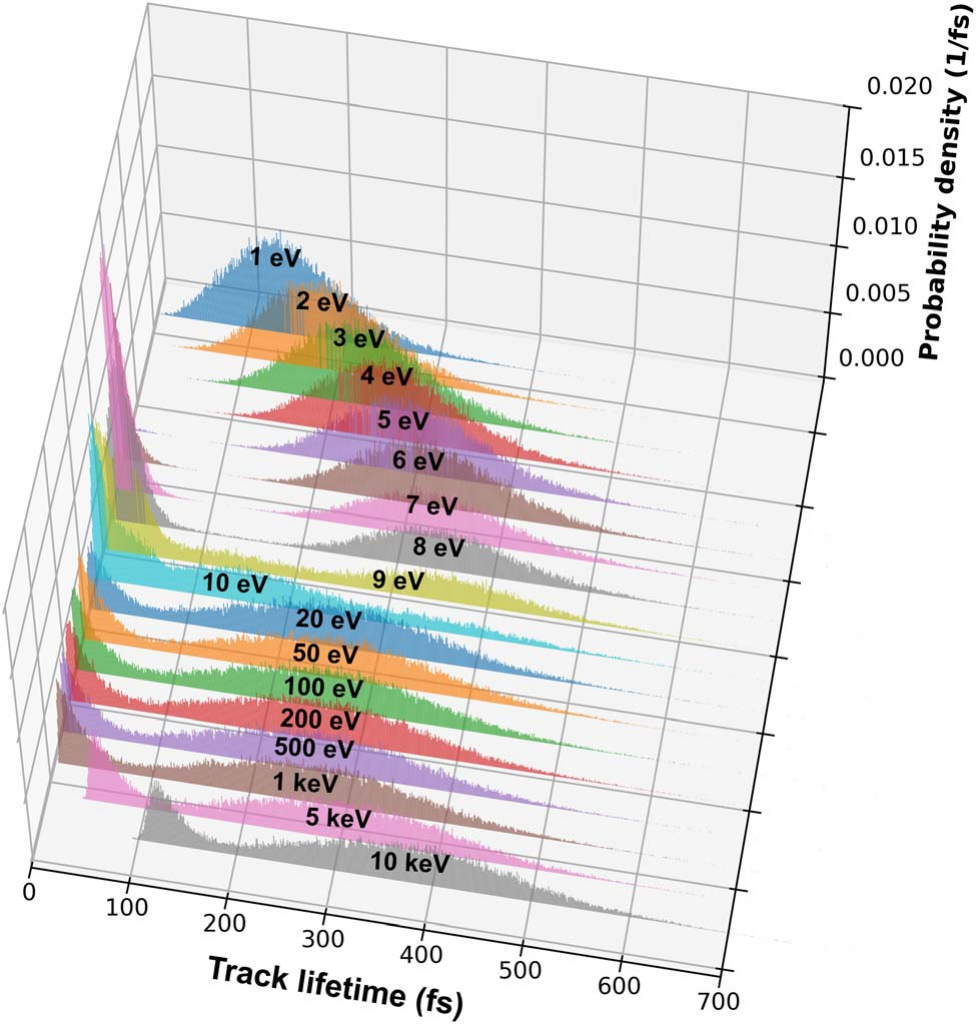
Distributions of mean track lifetime for electrons with *E*_0_ ranging from 1 eV to 10 keV. The labels denote the E_0_ of the electrons, highlighting the distinctly non-monotonic dependence of track lifetime on *E*_0_.

Fig. 10 presents the distribution of track lifetime as a function of *E*_0_ ranging from 1 eV to 10 keV. As evident from the figure, the dependence of track lifetime on *E*_0_ exhibits a distinctly non-monotonic behavior. Specifically, for electrons with *E*_0_ up to 5 eV, higher-energy electrons take longer to thermalize as their *E*_0_ increases. This is because vibrational and librational inelastic interactions transfer only small amounts of energy per scattering event. For electrons with *E*_0_ between 5 and 8 eV, the distributions evolve into well-separated bimodal forms, characterized by an additional peak at much shorter times. This short time peak originates from track-termination events driven by TNA mechanism, which is only operative for electrons above ∼4 eV (see Fig. 1 (c)). In terms of 〈*R*_gem_〉, this behavior corresponds to the local maximum observed in Fig. 6 around 5–6 eV, after which higher-energy electrons may travel shorter distances. However, not all electrons undergo TNA-induced termination; a fraction survives, continues to decelerate, and enters the sub-5 eV regime, giving rise to the longer-time peak. Electrons with *E*_0_ of 9–10 eV experience both TNA and significant energy loss *via* electronic excitation, and consequently this energy range marks a local minimum in the 〈*R*_gem_〉 curve. Above ∼10 eV, where electrons begin crossing the ionization threshold, the distribution evolves into a partially overlapping bimodal form, reflecting both the diminishing influence of TNA-driven processes and the onset of substantial energy loss through ionization. Up to approximately 1 keV, the increase in travel distance is largely compensated by the 
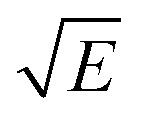
 scaling of electron velocity, resulting in an almost energy-independent track lifetime and a quasi-constant plateau. At 5 keV and higher, the fractional energy loss relative to the *E*_0_ continues to decrease, causing the electron's total travel distance to grow roughly in proportion to its *E*_0_. Although the electron speed also increases as 
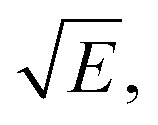
 moderating the rise in track lifetime, the distribution nonetheless shifts toward longer temporal regimes with a sublinear scaling trend, while retaining a bimodal character.

## 4. Conclusions

In this study, we developed a MC simulation framework to model electron scattering and transport phenomena in liquid water. By employing a comprehensive set of cross sections and incorporating energy loss and angular deflection schemes specific to each scattering process, the model reproduces the mean geminate separation distance, 〈*R*_gem_〉. This quantity is defined as an extension of the conventional thermalization distance, 〈*R*_th_〉, that explicitly incorporates EAD and autoionization processes. The simulated 〈*R*_gem_〉 values are consistent with photoinjection measurements at very low electron energies (<2.5 eV),^57^ with theoretical approximations—including reported CSDA values—up to 10 keV,^41,59–61^ and, importantly, with average spur radii of 6.9–8.3 nm reported in the literature,^52,53,104^ which were designed to fit radiolysis scavenging yields and picosecond transient absorbance experiments.

The validity of the ASW cross section data sets of Sanche and coworkers^21^ and the electronic energy loss sampling approach adopted herein was previously verified through successful reproduction of Pt–ASW EELS spectra.^31^ While those measurements were restricted to the limited LEE cases, the present work extends the analysis to a substantially broader energy range. In doing so, we further addressed concerns regarding the completeness of the ASW data by extrapolating their inelastic data sets, adopting recently measured liquid EMFP data, and scaling recommended gas-phase value for TNA formation near the resonance peak—thereby achieving consistency with available experimental observations across the relevant energy domain. We also demonstrated that ASW elastic cross section of Sanche and coworkers^21^ is not suitable for determining 〈*R*_gem_〉 of LEEs in liquid-phase water, particularly below 20 eV, even when substantial scaling is applied.

Our predicted 〈*R*_gem_〉 resolves the long-recognized discrepancies in reported average spur radii by relying on fewer and more physically constrained assumptions than earlier models. Moreover, by explicitly treating TNA and electron-impact excitation processes while accounting for ejection length and photoionization efficiency, we successfully reproduce the experimentally observed initial *G*-value of the pre-solvated electron on the sub-picosecond timescale.

A notable feature of this study is the systematic evaluation of multiple approximations and data sets, leading to what we believe are the most physically justified and predictive assumptions for modeling electron transport in liquid water. Nevertheless, several aspects remain unresolved, including uncertainties in the collision kinematics of electron-impact excitation, the treatment of unique LEE characteristics in the very low energies, challenges in the quantitative prediction of radiolytic species related to H_2_ formation, and the lack of reliable TNA cross sections in the liquid-phase water, which continue to be topics of active investigation. Hence, new experimental benchmarks, particularly those refining fundamental data sets for LEEs in liquid water, are critically needed to advance the physical interpretation of these processes in computational studies.

## Author contributions

H. L. developed the methodology, performed all simulations, and wrote the initial draft of the manuscript. D. M. B. and R. G. M. jointly conceived the study, provided theoretical and technical guidance, and supervised the project. All authors discussed the results and contributed to revising and finalizing the manuscript.

## Conflicts of interest

There are no conflicts to declare.

## Supplementary Material

RA-016-D6RA00710D-s001

RA-016-D6RA00710D-s002

RA-016-D6RA00710D-s003

## Data Availability

All data supporting the findings of this study, including simulation parameters, cross-sections, and data sets used to generate the figures, are available within the article and supplementary information (SI). Supplementary information: additional figures, comprehensive simulation details, input parameters, and all raw data generated and analyzed in this study, ensuring full transparency and reproducibility of the reported results. See DOI: https://doi.org/10.1039/d6ra00710d.
