# Supplementary material for: Electron transport (0.2 eV–10 keV) in liquid water: resolving discrepancies between track simulations and radiolysis data
Source: RSC Adv. 2026 Apr 17;16(22):20065–86. doi: 10.1039/d6ra00710d (PMC13088954; doi:10.1039/d6ra00710d)
Supplement: RA-016-D6RA00710D-s003 [file RA-016-D6RA00710D-s003.pdf]

Supplementary Information (SI) for RSC Advances.
This journal is © The Royal Society of Chemistry 2026

Supplementary Information

Electron Transport (0.2 eV–10 keV) in Liquid Water: Resolving Discrepancies Between Track Simulations and Radiolysis Data

Hoon Lee [a,b], David M. Bartels [a,c], Ryan G. McClarren [b,*]

[a] *Notre Dame Radiation Laboratory, University of Notre Dame, Notre Dame, Indiana 46556, United States*
[b] *Department of Aerospace and Mechanical Engineering, University of Notre Dame, Notre Dame, Indiana 46556, United States*
[c] *Department of Chemistry and Biochemistry, University of Notre Dame, Notre Dame, Indiana 46556, United States*

* Correspondance : rmcclarr@nd.edu

Table of Contents

1. Vibrational inelastic energy loss
2. Collision frequency
3. Ionization energy loss sampling
4. Modified Born-Ochkur exchange correction
5. $\langle R_{gem} \rangle$ comparison with OpenTOPAS
6. Secondary electron energy spectrum
7. Spur size and $\langle R_{gem} \rangle$

1. Vibrational inelastic energy loss

The amount of kinetic energy lost by an electron via inelastic collisions that excite the intermolecular vibrational and librational modes of water were treated using the integral cross sections reported from electron energy loss spectroscopy (EELS) experiments of Sanche and coworkers on amorphous solid water (ASW) [1]. These measurements resolve the major low energy loss features of condensed phase water, including the translational phonon bands (v'_T, v''_T: ~10–24 meV), the librational modes (v'_L, v''_L: ~61–92 meV), the bending vibration (v_2: ~204 meV), the stretching modes ($v_{1,3}$, v_3: ~417–460 meV), and the combination ($v_{1,3}$+v_L: ~500 meV) and overtone of the stretching modes (2($v_{1,3}$): ~ 835 meV), each of which contributes a characteristic amount of energy loss.

The detailed spectral shape of the EELS data depends on E_0 and experimental geometry, such as the incident and detection angles. Nevertheless, the relative ordering and characteristic energy scales of the inter- and intra-molecular excitation modes remain robust [1]. This consistency is further supported by direct quantitative comparison with dielectric-function–derived quantities, including the single differential cross section (SDCS) of Gadeyne et al. [2] and the energy loss function (ELF) reported by Kai et al. [3], as shown in Figure S1, despite the fact that these quantities represent fundamentally different physical observables. Because our simulation explicitly employs the nine mode-resolved ASW cross sections, it therefore incorporates experimentally validated condensed phase scattering probabilities, ensuring that the modeled vibrational and librational energy loss events remain physically consistent with experimental observations.

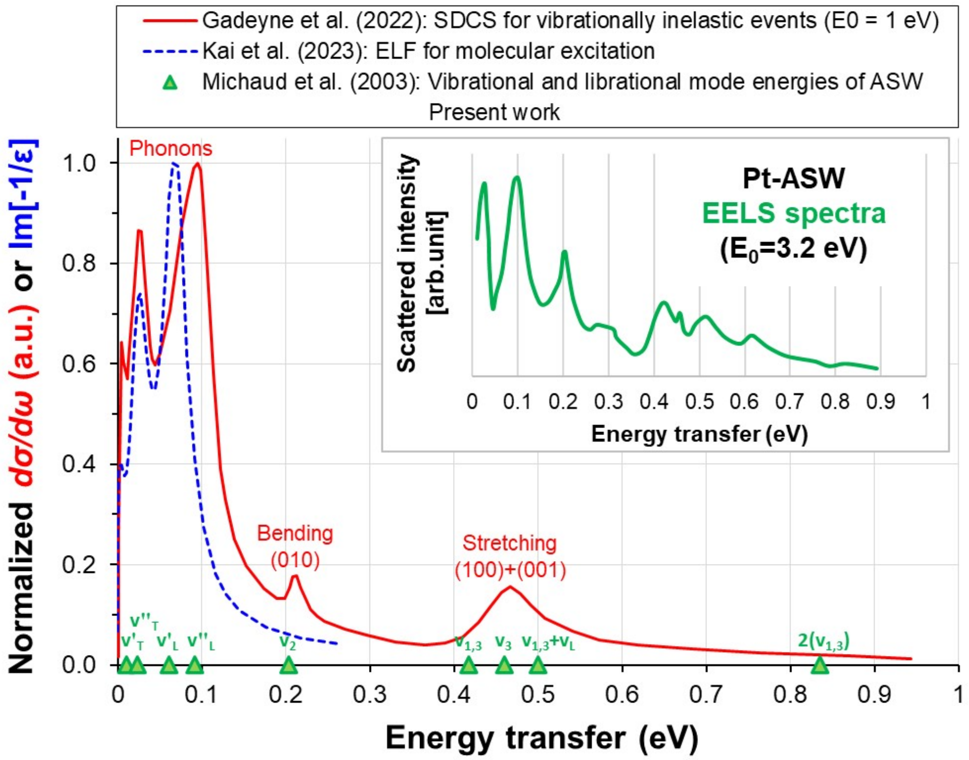

Figure S1. Vibrational inelastic energy loss distributions. Solid red: SDCS for vibrationally inelastic events at E_0= 1 eV [2], Dashed blue: ELF corresponding to the molecular excitation energy absorption efficiency [3], Green triangles: peak energies of the vibrational excitation modes of ASW [1] adopted in present work. Inset: Pt–ASW EELS result for E_0 = 3.2 eV case [4]. Zero-loss peak omitted for clarity.

2. Collision frequency

Figure S2 shows the frequency of various types of interactions experienced by electrons with different initial (incident) energy, E_0, (0.2 eV–10 keV) during the transport process. As shown, elastic scattering is the most frequent interaction across the entire energy range due to its large cross section and negligible energy loss character.

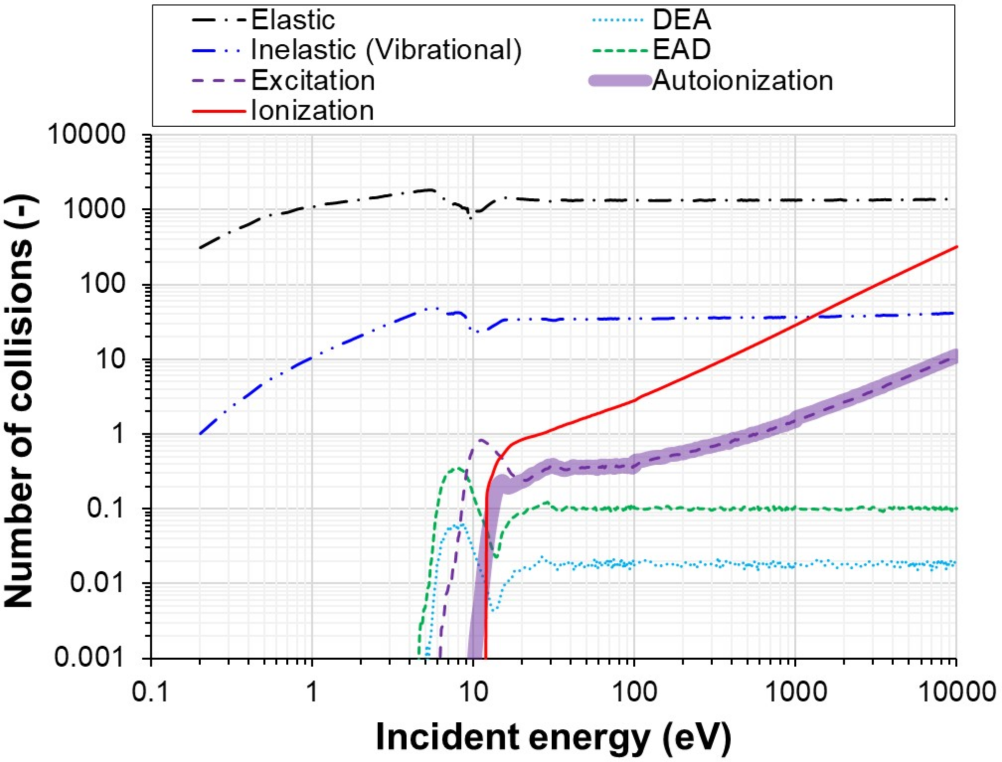

Figure S2. Collision frequencies for each type of scattering event experienced by electrons with initial kinetic energies between 0.2 eV and 10 keV, averaged over 10^4 realizations.

Both elastic and inelastic (vibrational) collisions exhibit an increasing trend with E_0 at very low energies, followed by a sharp drop near ~10 eV, after which their frequencies remain essentially independent of E_0. A similar pattern is observed for dissociative electron attachment (DEA) and electron autodetachment (EAD) processes. This behavior arises because electronic events—particularly ionization, which induces large energy losses—become much more dominant at higher energies, and very few events are required to reach the low energy regime. Consequently, high-energy electrons rapidly lose energy and fall into the ~10–20 eV regime. As estimated from the total track lifetime results (see Figure 10 in the main text), the time required for an electron's energy to decrease to near the ionization threshold remains nearly constant over the 20 eV–1 keV E_0 range due to compensating effects between travel distance and electron velocity.

Most elastic and vibrational collisions occur within the low energy region (< 20 eV), and transient negative anion (TNA) driven processes also influence track early termination predominantly in the 5–15 eV range. This is also the reason why the largest $\langle R_{gem} \rangle$

occurs at ~5 eV (see Figure 1 (c) and Figure 6 in the main text), where vibrational inelastic scattering dominates the energy loss channels and electronic events are energetically forbidden. Ultimately, the increasing contribution of ionization causes the frequencies of all other interactions to become nearly independent of E_0 above ~20 eV, resulting in the observed plateau behavior.

The frequency of electronic excitation events peaks around ~11 eV, decreases, and then gradually increases again. This trend reflects the onset of ionization at the ionization potential (set to 11.16 eV in this work), after which a small fraction of excitation events (about 5%) persists even for E_0 exceeding 100 eV (see Figure 5 (b) in the main text).

The ionization frequency increases monotonically with E_0. At 10 keV, the ionization frequency reaches nearly 300, implying the generation of ~300 secondary electrons, on average, during the transport of a 10 keV primary electron. Additional secondary electrons may be produced via autoionization from neutral excited water molecules (H_2O^*). The relative contribution of this pathway follows the product of the impact-excitation frequency and the energy-dependent photoionization efficiency. Once this value approaches unity above ~20 eV (see Figure 6 of Ref. [5]), the autoionization contribution becomes indistinguishable from excitation. Compared to electron-impact ionization, autoionization accounts for ~1/30 of the ionization frequency in the 1–10 keV range. However, this value is still more than one to two orders of magnitude higher than the TNA-driven DEA frequency, which remains at ~0.02. The low DEA frequency obtained in this work is consistent with scavenging measurements of molecular hydrogen formation in water radiolysis [6] and confirms the plausibility of the TNA cross section assumed in this work, which is constructed as the sum of the DEA and EAD cross sections.

3. Ionization energy loss sampling

Figure S3 (a-b) shows the distributions of ionization energy loss as a function of the binding energy, Q, and scattering cosine, μ. Each point represents a single electronic event, color-coded by the incident electron energy, E_0. The histogram at the top represents the distribution of sampled Q, revealing distinct contributions from the $1b_1$, $3a_1$, $1b_2$, and $2a_1$ molecular-orbital regions, while the histogram on the right shows the total ionization energy loss distribution integrated over all events. For E_0 above 100 eV (Figure S3 (a)), the minimum and maximum allowable energy losses are set by Q and by classical collision kinematics [7], respectively. For E_0 at or below 100 eV (Figure S3 (b)), the energy loss is first sampled directly from the single differential cross section (SDCS); based on this energy loss, Q is then sampled from the density of state (DOS) distribution. In all cases, the kinetic energy of the secondary electron is given by the difference between the sampled energy loss and the corresponding Q.

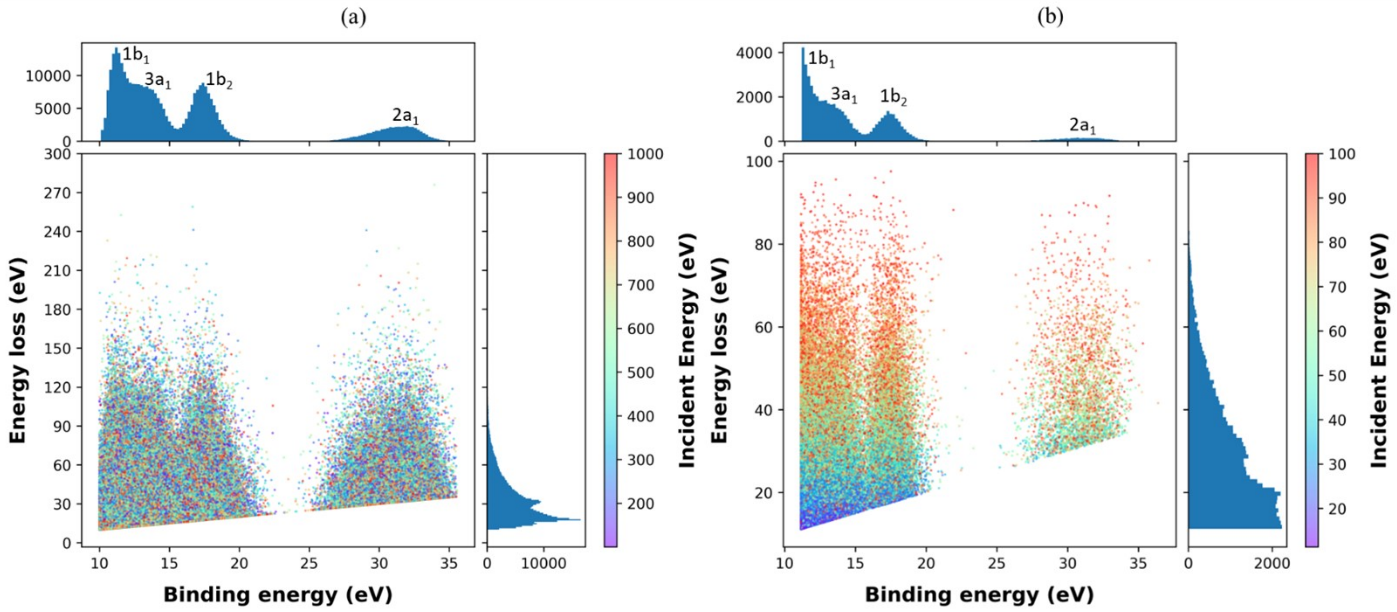

Figure S3. Binding energy, Q, and ionization energy loss distributions for (a) E_0 > 100 eV cases, and (b) E_0 ≤ 100 eV cases.

4. Modified Born-Ochkur exchange correction

In this work, for electrons energies below 100 eV, we adopt the energy loss function (ELF) data sets derived using the random phase approximation (RPA) method [8]. These data are reported to offer improved accuracy compared to previously reported Mermin–Penn algorithm (MPA) based ELF data sets [9], particularly in the high-k region where the MPA approach relies on model-dependent extrapolation of $k = 0$ data. By providing a first-principles description of the full k- and ω-dependent ELF, including additional high momentum transfer structures that are consistent with experimental inelastic X-ray scattering (IXS) measurements, the RPA-derived data yields a more faithful representation of the underlying excitation physics.

The RPA-ELF exhibits two notable features that distinguish it from the MPA-ELF. First, it shows a secondary peak (~30 eV) that emerges in the higher-k region from excitation of the $2a_1$ electrons. Second, the energy loss distribution becomes non-zero before the excitation threshold, with finite intensity appearing at energies around 4.5 eV (see Figure 3 and the related discussion in Ref. [8]). Because the single differential cross section (SDCS) is obtained by integrating the 3-D ELF surface over the physically allowed momentum transfer range, the resulting SDCS inevitably inherits a double peak structure, as shown in Figure S4. For the SDCS used in the present work (solid red), it also leads to enhanced energy loss intensity in both the low- (< 20 eV) and high-energy (> 30 eV) regions compared to the MPA-ELF at $k = 0$ (i.e., optical energy loss function (OELF)).

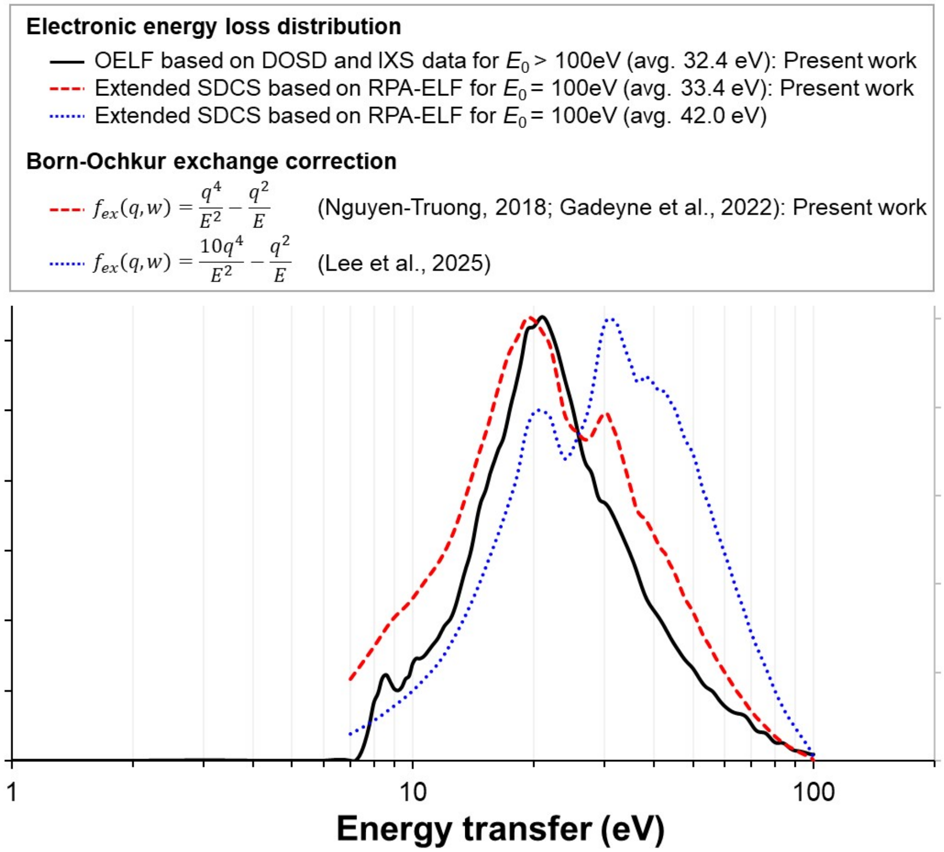

Figure S4. Electronic energy loss distributions: (i) the OELF (black) used for high energy cases (E_0 > 100 eV), and the RPA-ELF derived SDCS obtained using the modified Born-Ochkur correction schemes reported in (ii) Refs. *[2, 9]* (red), and (iii) Ref. *[10]* (blue). The RPA-ELF derived SDCS exhibits a double peak structure, with a secondary peak appearing near 30 eV. In the SDCS used in the present work, the intensity is enhanced in both the < 20 eV and > 30 eV regions compared to the MPA-based OELF, while the mean energy loss remains nearly identical (32 eV vs 33 eV).

Figure S5 (a–d) shows that the magnitude of the secondary peak can be adjusted to some extent by applying the Born–Ochkur correction and its modified forms [2, 8, 9]. However, the peak cannot be fully removed, and because its contribution is accumulated through the integration step, a certain degree of deviation from the OELF shape inevitably persists, regardless of the chosen crossover criterion (currently set to 100 eV). This discrepancy may also be partly attributed to the extrapolation procedure used to extend the momentum transfer range of the RPA-ELF data sets. In this work, we extend the RPA-ELF from its original range (from 1.32 to 6.0 bohr^{-1}) using a simple exponential-decay assumption; this approach may not accurately capture the true high k - behavior, which could be more complex. Fully resolving this would require computationally demanding first-principles calculations.

For reference, the MPA-ELF data set reported in Ref. [8], which is based on the OELF, is also generated using an extrapolation scheme. In that case, the OELF is extended to finite k by shifting and broadening a set of fitted Lorentzian poles—an empirical procedure that reproduces the k-dependent damping of the main peak but cannot generate new high-k structures, such as the secondary peak that emerges in first-principles RPA calculations. Ref. [9] argues that this secondary structure arises naturally in RPA/time-dependent density-functional theory (TDDFT)-based ELF calculations, because these approaches explicitly capture high-k excitation physics such as plasmon damping and interband transitions. As a result, they show much better agreement with IXS measurements than OELF- or

MPA-based models. In contrast, in the present work, electronic energy loss in the high-energy regime (E_0 > 100 eV) is sampled from the OELF. To minimize inconsistencies between the low- and high-energy regions and to maintain physical continuity across the crossover, we therefore apply the Born–Ochkur modification in a way that suppresses the secondary peak dominance and preserves the 20 eV main peak shape. At present, the true high-k ELF peak structure in the several-tens-of-eV or higher regime, as well as the appropriate form of Born–Ochkur correction in the low-energy dielectric formalism, both appear to be unsolved issues.

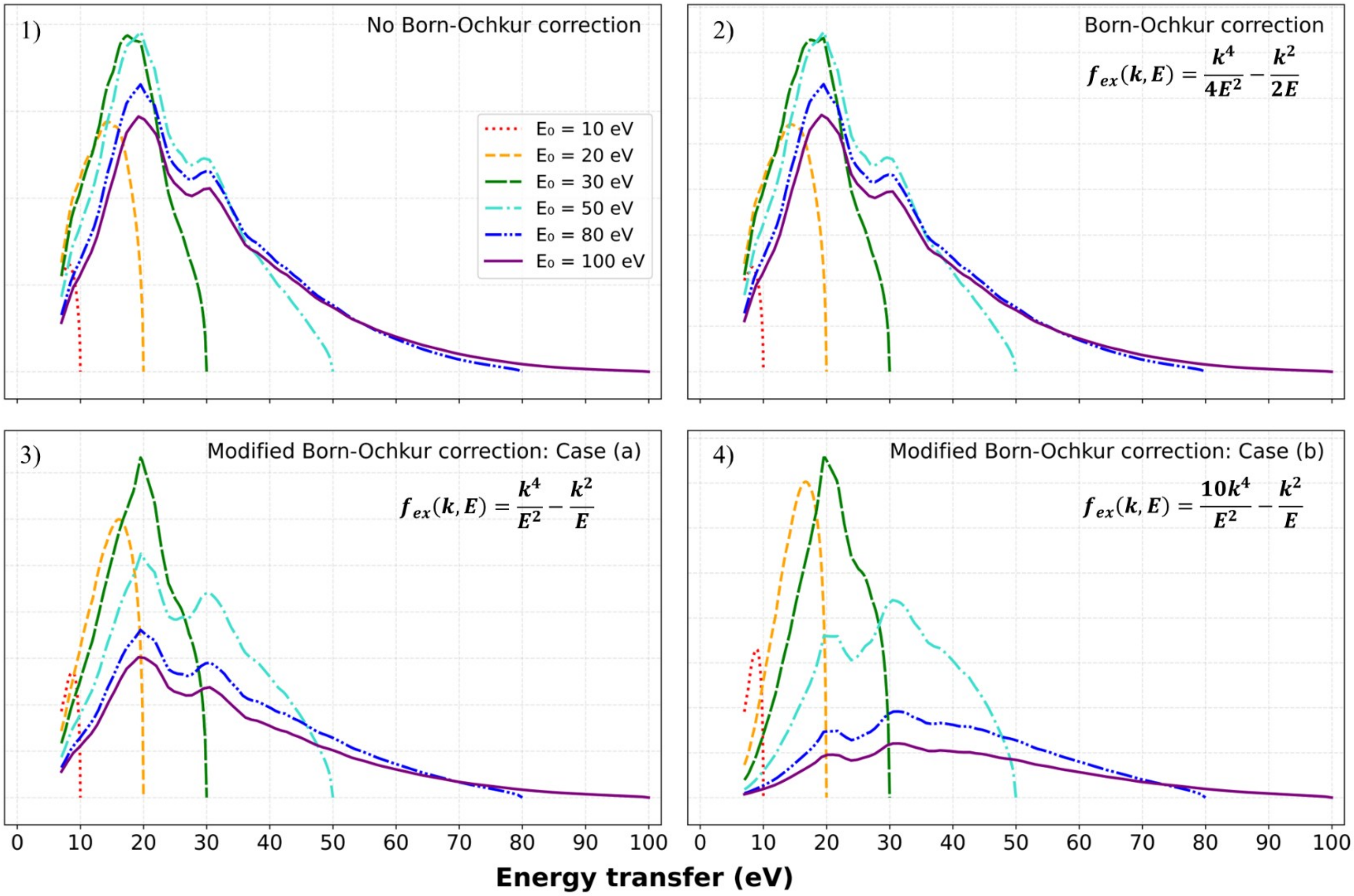

Figure S5. Effect of the Born–Ochkur correction and its modified forms on the shape of the SDCS. Shown are the SDCS computed 1) without applying the correction, 2) with the correction, and with the modified corrections, 3) Case (a), and 4) Case (b). Modifications adjust the magnitude of the high-k contribution in different ways: in Case (a), the 20 eV peak remains the dominant feature, whereas in Case (b), the stronger enhancement of the exchange term produces a more pronounced alteration of the peak structure.

Importantly, as discussed in the main text, these shape differences between the SDCS and the OELF have a negligible influence on high energy transport properties such as $\langle R_{gem} \rangle$ (or $\langle R_{th} \rangle$). Threshold-sensitive quantities, such as TNA-driven channels, could, in principle, be affected if the SDCS shape altered the intensity exactly at the threshold. However, in our model, both DEA and EAD are treated as separate processes with their own cross sections in the 5–15 eV range, rather than being embedded within the electronic interactions. Consequently, the TNA-related radiolytic species yield is unaffected by the choice of Born–Ochkur correction or by the detailed form of the SDCS.

5. $\langle R_{gem} \rangle$ comparison with OpenTOPAS

To provide an independent numerical reference, $\langle R_{gem} \rangle$ was recalculated using a 7 eV cutoff so that the present model could be compared directly with the low energy electron (LEE) transport implemented in OpenTOPAS (v4.0), which incorporates the widely used GEANT4-DNA physics for electron slowing-down in liquid water. As shown in Figure S6, the agreement between the present model and OpenTOPAS at E_0 above ~200 eV is expected because $\langle R_{gem} \rangle$ in this regime are governed primarily by cumulative inelastic stopping rather than the details of sub-excitation scattering; thus, the consistency serves as a useful cross-check that our cross sections reproduce the correct high-energy attenuation behavior. For context, Figure S6 also includes several historical theoretical and CSDA-based estimates [11, 12, 13, 14, 15] (symbols), as well as earlier Monte Carlo (MC) track calculations [16] (dashed purple). Including these data sets illustrates how improvements in the underlying cross sections and energy-loss descriptions evolved over time and highlights the spread among pre-existing predictions. Notably, while the present model is qualitatively consistent with the OpenTOPAS results across the full energy range, it shows quantitatively closer agreement with the CSDA-based estimates, supporting the robustness of the cross-section framework, including the DEA-channel scaling and the extrapolation procedure used to extend the ASW data sets.

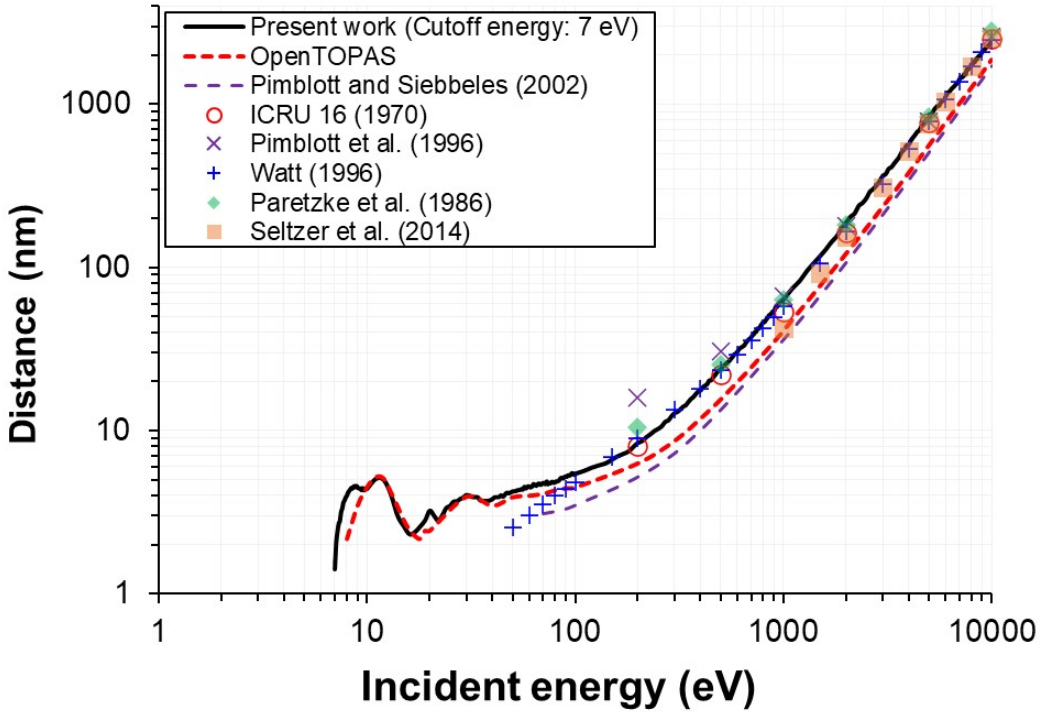

Figure S6. Comparison of mean geminate separation distance, $\langle R_{gem} \rangle$, between the present work—where the cutoff energy in the simulation was increased from 0.2 eV to 7 eV—and the OpenTOPAS (v4.0) simulation results.

6. Secondary electron energy spectrum

To calculate the spatial extent of spur in low linear energy transfer (LET) radiolysis, we require the energy spectrum of secondary electrons, together with the determined cross section data sets, energy loss, and angular deflections for each collision (Recall from Figure

S2 that a single 10 keV electron generates ca. 300 secondaries). Determining this entry spectrum is critical for accurately predicting the total energy loss, spatial distribution, and product yields of species arising from spur chemistry. However, similar to the other parameters described earlier, a review of the literature reveals that various forms of secondary electron energy spectra [2, 17, 18, 19] have been proposed, with no solid consensus yet established.

In this work, we simulated five representative spectra, shown in Figure S7 (a), including model results from OpenTOPAS [20] and the present work, to identify and analyze their effects on the predicted $\langle R_{gem} \rangle$ of secondary electrons. In our work, the energy of secondary electrons was calculated by subtracting the binding energy, Q, from the ionization energy loss. It should be noted that the only available experimental data sets, the vacuum ultra violet (VUV) photoelectron spectrum [2] (shown in green), essentially represents a low energy tail spectrum that includes not only true secondary electrons but also primary electrons that have lost substantial energy through inelastic collisions. As pointed out in Ref. [21], the term "secondary electron energy distribution" is defined differently across research fields. In this study, we included these data sets as one of the input spectra to comparatively evaluate how different spectral shapes influence the calculated $\langle R_{gem} \rangle$. Figure S7 (b) shows the distribution of $\langle R_{gem} \rangle$ of the secondary electrons associated with each distribution. A pronounced bimodal distribution is observed, which primarily arises from the strong weighting of all input energy spectra toward low energies, with a most probable energy around 1 eV. In particular, for the two distributions with higher mean energies—Ref. [18] (orange) and the present work (purple)—the low $\langle R_{gem} \rangle$ peak becomes more prominent, as these spectra are more strongly exposed to track termination via TNA-driven mechanisms.

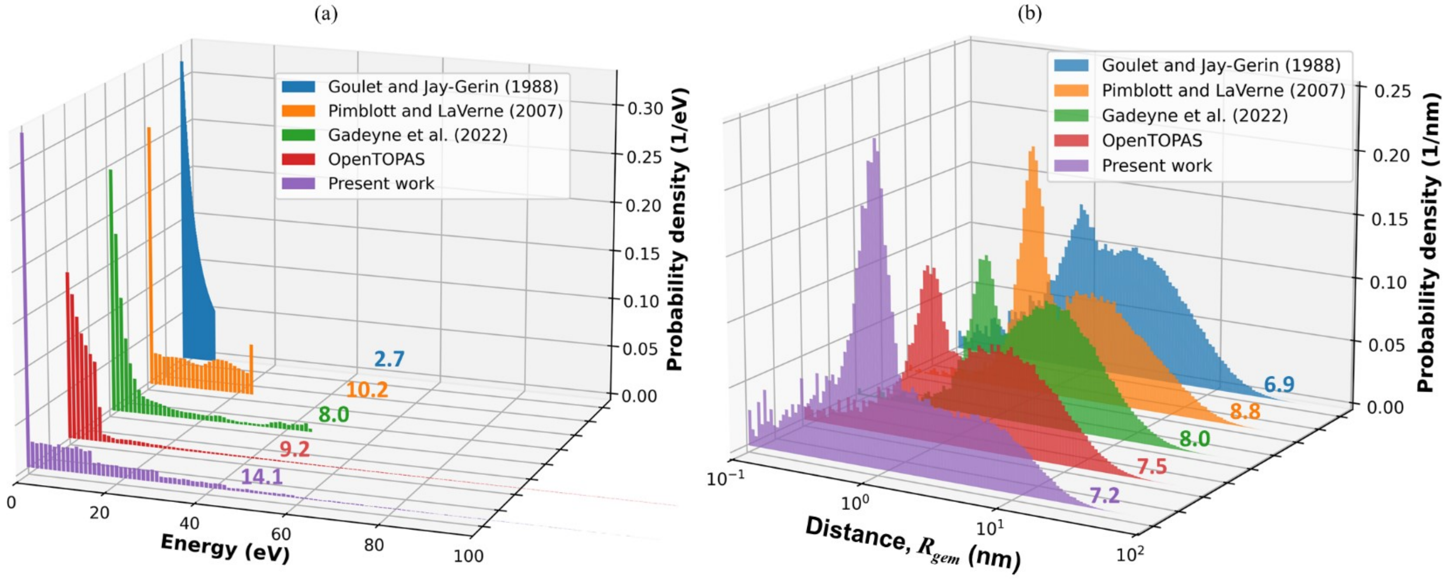

Figure S7. (a) E_0 distribution of secondary electrons used in the simulations, obtained from quantitative analyses *[17, 19]*, track simulations *[18]*, liquid microjet experiments *[2]*, and the OpenTOPAS *[20]*. The OpenTOPAS (red) and present work (purple) distributions correspond to incident electron energies of 1 keV and 10 keV, respectively. The numbers indicate the mean energy (eV) of each distribution. (b) Geminate separation distance distribution of secondary electrons obtained from the simulations. The number indicates the mean value of each distribution.

7. Spur size and $\langle R_{gem} \rangle$

To assess the performance of the track-structure simulation model, the mean geminate separation distance, $\langle R_{gem} \rangle$, calculated from the secondary electron energy distribution in Figure S7 (b) is compared with other theoretical approximations and available experimental measurements, as illustrated in Figure S8. As discussed in the main text, direct experimental data for $\langle R_{gem} \rangle$ (or $\langle R_{th} \rangle$) are available only at very low E_0 below 2.5 eV [22, 23] (blue bars: h) and i)). These direct photoinjection measurements provide the distance as a function of E_0, yielding a range of values rather than a single characteristic distance. At higher energies, $\langle R_{gem} \rangle$ must therefore be inferred from modeling.

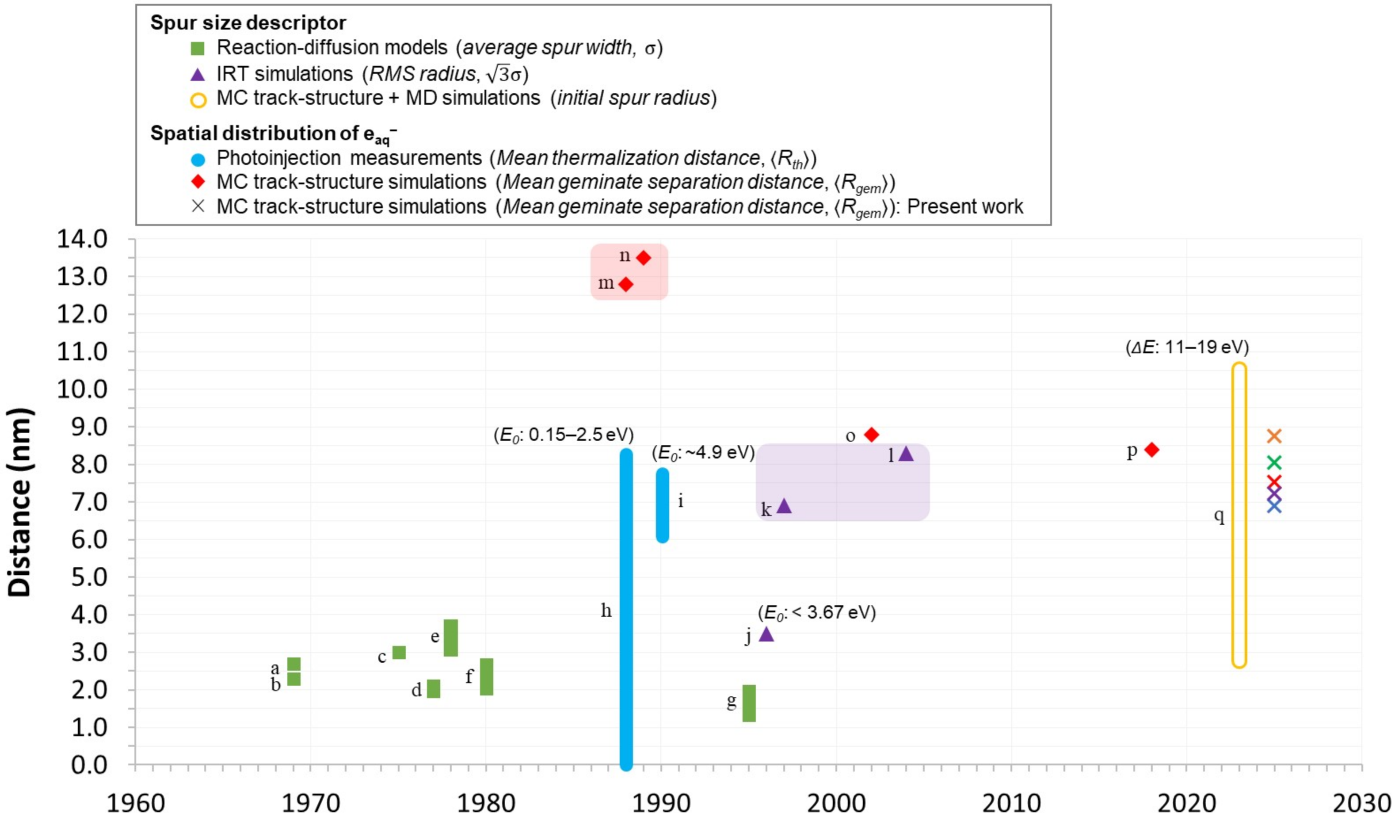

Figure S8. Chronological summary of reported spur size descriptors and $\langle R_{gem} \rangle$ among analytical estimates, IRT-based stochastic simulations, MC track-structure calculations, and hybrid MC-MD approaches. Present work (×): Calculated with secondary electron energy spectra (see Figure S7 (a)) adopted from blue) *[17]*; orange) *[18]*; green) *[2]*; red) *[20]*; purple) present model. Deterministic diffusion-reaction models: a) *[24]*; b) *[25]*; c) *[26]*; d) *[27]*; e) *[28]*; f) *[29]*; g) *[30]*. Experimental measurements: h) *[22]*; i) *[23]*. IRT-based stochastic simulations: j) *[31]*, k) *[32]*; l) *[33]*. MC track-structure simulations: m) *[17]*; n) *[34]*; o) *[35]*; p) *[36]*. MC-MD hybrid simulation: q) *[37]*. The two shaded regions indicate the discrepancies between the IRT (purple) and full-MC (red) results.

Early studies predominantly employed deterministic approaches based on reaction–diffusion kinetics [24, 25, 26, 27, 28, 29, 30], assuming that the local spatial distribution of e_{aq}^- follows a spherical 3-D Gaussian profile. The data shown as green squares (a)–g)) represent the standard deviation, σ, of the distribution, also referred to as “spur width.” These models typically relied on fitting analytical solutions to experimental radiolysis data, using the time-dependent yield of e_{aq}^- and concentration-dependent scavenger yields as a benchmark. With increasing computational capabilities, more realistic stochastic simulations became feasible, including explicit MC

track-structure models [17, 34, 35, 36] (red diamonds: m)–p)), which trace individual electron trajectories and explicitly evaluate transport and scattering processes. Independent reaction time (IRT)–based methods [31, 32, 33] (purple triangles: j), k), l)) instead determine diffusion–reaction outcomes probabilistically without explicit time-resolved particle tracking, in which R_{gem} is treated not as a directly calculated physical observable but as a model parameter, typically adjusted—together with recombination probabilities—through global fitting to reproduce early-time radiolysis yields such as G(e_{aq}^-). The data shown as purple triangles (k)–l)) represent the root-mean-square (RMS) radius of the distribution, $\sqrt{3}\sigma$ (≈1.7σ), also referred to as "spur radius." More recently, MC–molecular dynamics (MD) hybrid simulations have also reported spur radii [37] (yellow ellipse: q)), expressed as ranges dependent on the deposited energy, ΔE.

Among the previous theoretical predictions, we consider the later IRT results—particularly the value of 8.3 nm reported in Ref. [33] (l))—to provide the most reasonable benchmark. This estimate incorporated methodological refinements aimed at reproducing more recent picosecond laser measurements, leading to an increase relative to the earlier 1997 IRT value of 6.9 nm [32] (k)). The 8.3 nm value therefore represents a more appropriate reference and remains consistent with photoinjection measurements below 2.5 eV. Nevertheless, as shown, substantial discrepancies have persisted for decades, most notably between MC track-structure calculations and IRT-based predictions (two shaded regions: m) and n) vs. k) and l)).

It should be noted that the two MC track-structure simulations [35, 36] (o) and p)) that yield values comparable to the IRT predictions rely on strong and physically questionable assumptions. One such assumption is the *ad hoc* upscaling (×2) of ASW cross sections, introduced in an attempt to mitigate the discrepancies [35, 38, 39]. As previously pointed out [40], however, such scaling lacks a physical rationale and therefore cannot be regarded as a satisfactory approach.

Another assumption, supported by only a very limited body of literature, involves the introduction of additional low energy loss channels composed of two combined processes: electron capture at very low energies (below 1.5 eV) and DEA near about 10 eV [36, 41]. To construct the corresponding cross sections, charge-trapping data measured for isolated water clusters condensed onto thin films of Kr and Xe [42] have been employed. We do not consider this approach compelling for bulk liquid water for several reasons. First, the lower-energy peak attributed to energetic electron capture exhibits cross section values as large as about 0.1 nm^2, which leads to unrealistically small predicted $\langle R_{gem} \rangle$ [36] and fails to reproduce photoinjection measurements [22] near and below 1 eV. Second, the higher-energy peak attributed to DEA is also excessively large, reaching ~50% of the total scattering cross section [41] or dominating the most probable events [36] at 10 eV. This directly results in a pronounced minimum in the predicted $\langle R_{gem} \rangle$ at 10 eV (see Figures 2 and 4 of [41] and Figure 6 in the main text). Third, as a consequence of these assumptions, the resulting DEA frequency becomes exceptionally large: approximately 22.5 % of all interactions at an E_0 of 100 eV, increasing to nearly 90% [36] at an E_0 of 10 eV. Moreover, the fraction associated with the H^- channel alone exceeds 10%, which is not consistent with estimates inferred from scavenging experiments of H_2 formation in water radiolysis [6], if the branching ratios of DEA and EAD assumed in the model are accurate. By contrast, in the present work, the predicted DEA contribution remains below 2% (see Figure S2). Taken together, these

observations suggest that trapping mechanisms in bulk liquid water are expected to differ substantially from those operative in thin films of isolated water clusters. The condensed phase geometry in cluster-in-matrix experiments is fundamentally different from that of bulk liquid water, where earlier studies generally assumed that electrons become trapped in transient voids or cavities formed between water molecules [43, 44, 45], an interpretation supported by recent ab initio molecular dynamics simulations of the hydrated electron [46]. While such studies provide useful qualitative insight, the exceedingly large capture and DEA contributions implied by cluster-based cross sections indicate that these mechanisms cannot be directly transposed to liquid water.

Addressing these issues constitutes the central motivation of the present work, as no MC track simulation since the 1980s has reconciled the IRT vs. full–MC discrepancies without invoking such questionable assumptions. The present model predicts $\langle R_{gem} \rangle$ values ranging from 6.9 to 8.8 nm (five '×' symbols), depending on the choice of entry spectrum used as a modeling parameter (see Figure S7 (a)). Importantly, irrespective of the specific spectrum employed, the predicted values consistently fall within—or very close to—the IRT simulation results (k) and l)), thereby effectively resolving the long-standing discrepancy between IRT and MC track-structure predictions of $\langle R_{gem} \rangle$.

REFERENCES

[1] M. Michaud, A. Wen and L. Sanche, "Cross sections for low-energy (1–100 eV) electron elastic and inelastic scattering in amorphous ice," *Radiation research,* vol. 159, pp. 3-22, 2003.

[2] T. Gadeyne, P. Zhang, A. Schild and H. J. Wörner, "Low-energy electron distributions from the photoionization of liquid water: a sensitive test of electron mean free paths," *Chemical Science,* vol. 13, no. 6, pp. 1675-1692, 2022.

[3] T. Kai, T. Toigawa, Y. Matsuya, Y. Hirata, T. Tezuka, H. Tsuchida and A. Yokoya, "Initial yield of hydrated electron production from water radiolysis based on first-principles calculation," *RSC advances,* vol. 13, no. 11, pp. 7076-7086, 2023.

[4] M. Michaud and L. Sanche, "Absolute vibrational excitation cross sections for slow-electron (1–18 eV) scattering in solid H2O," *Physical Review A,* vol. 36, no. 10, pp. 4684-4699, 1987.

[5] K. Tan, C. Brion, P. Van der Leeuw and M. Van der Wiel, "Absolute oscillator strengths (10–60 eV) for the photoabsorption, photoionisation and fragmentation of H2Os," *Chemical Physics,* vol. 29, no. 3, pp. 299-309, 1978.

[6] M. Sterniczuk and D. Bartels, "Source of molecular hydrogen in high-temperature water radiolysis," *The Journal of Physical Chemistry A,* vol. 120, no. 2, pp. 200-209, 2016.

[7] J. K. Shultis and R. E. Faw, Fundamentals of Nuclear Science and Engineering, New York: Marcel Dekker, Inc., 2002.

[8] H. Nguyen-Truong, Z. Hu and B. Da, "Low-Energy Electron Inelastic Mean Free Path for Liquid Water," *The Journal of Physical Chemistry Letters,* vol. 16, no. 18, pp. 4555-4562., 2025.

[9] H. Nguyen-Truong, "Low-energy electron inelastic mean free paths for liquid water," *Journal of Physics: Condensed Matter,* vol. 30, no. 15, p. 155101, 2018.

[10] H. Lee, D. Bartels and R. McClarren, "Toward a Comprehensive Understanding of Low-Energy Electron Energy Loss Spectra of Amorphous Ice," *The Journal of Physical Chemistry Letters,* vol. 16, pp. 12068-12073, 2025.

[11] "ICRU Report 16: Linear Energy Transfer," ICRU Publications, Washington D.C., 1970.

[12] C. Tung, J. Lou and S. Lin, "Pathlength distributions of low energy electrons in water," *Nuclear Instruments and Methods in Physics Research Section B: Beam Interactions with Materials and Atoms,* vol. 16, no. 1, pp. 83-87, 1986.

[13] H. Paretzke, J. Turner, R. Hamm, H. Wright and R. Ritchie, "Calculated yields and fluctuations for electron degradation in liquid water and water vapor," *The Journal of chemical physics,* vol. 84, no. 6, pp. 3182-3188, 1986.

[14] S. Pimblott, J. LaVerne and A. Mozumder, "Monte Carlo simulation of range and energy deposition by electrons in gaseous and liquid water," *The Journal of Physical Chemistry,* vol. 100, no. 20, pp. 8595-8606, 1996.

[15] S. Seltzer, J. Fernandez-Varea, P. Andreo, P. Bergstrom, D. Burns, I. Krajcar Bronić, C. Ross and F. Salvat, "Key data for ionizing-radiation dosimetry: measurement standards and applications," ICRU Report 90, 2016.

[16] S. Pimblott and L. Siebbeles, "Energy loss by non-relativistic electrons and positrons in liquid water," *Nuclear Instruments and Methods in Physics Research Section B: Beam Interactions with Materials and Atoms,* vol. 194, no. 3, pp. 237-250, 2002.

[17] T. Goulet and J. Jay-Gerin, "Thermalization distances and times for subexcitation electrons in solid water," *The Journal of Physical Chemistry,* vol. 92, no. 24, pp. 6871-6874, 1988.

[18] S. Pimblott and J. LaVerne, "Production of low-energy electrons by ionizing radiation," *Radiation Physics and Chemistry,* vol. 76, pp. 1244-1247, 2007.

[19] R. Platzman, "Subexcitation electrons," *Radiation Research,* vol. 2, no. 1, pp. 1-7, 1955.

[20] The TOPAS Collaboration, [Online]. Available: https://github.com/OpenTOPAS. [Accessed 2024].

[21] S. Malerz, F. Trinter, U. Hergenhahn, A. Ghrist, H. Ali, C. Nicolas, C. Saak, C. Richter, S. Hartweg, L. Nahon and C. Lee, "Low-energy constraints on photoelectron spectra measured from liquid water and aqueous solutions," *Physical Chemistry Chemical Physics,* vol. 23, no. 14, pp. 8246-8260, 2021.

[22] V. V. Konovalov, A. M. Raitsimring and Y. D. Tsvetkov, "Thermalization lengths of “subexcitation electrons” in water determined by photoinjection from metals into electrolyte solutions," *International Journal of Radiation Applications and Instrumentation. Part C. Radiation Physics and Chemistry,* vol. 32, p. 623–632, 1988.

[23] V. Konovalov and A. Raitsimring, "Photoelectron injection and additional photochemical processes on irradiation of the mercury—water interphase at 193 nm," *Chemical physics letters,* vol. 171, no. 4, pp. 326-334, 1990.

[24] A. Mozumder, "Neutralization of isolated ion pair in polar media. I. Theory for the yield of solvated electrons," *The Journal of Chemical Physics,* vol. 50, no. 8, pp. 3153-3161, 1969.

[25] H. Schwarz, "Applications of the spur diffusion model to the radiation chemistry of aqueous solutions," *The Journal of Physical Chemistry,* vol. 73, no. 6, pp. 1928-1937, 1969.

[26] A. Mozumder and J. Magee, "The early events of radiation chemistry," *International Journal for Radiation Physics and Chemistry,* vol. 7, no. 2-3, pp. 83-93, 1975.

[27] W. Burns, R. May, G. Buxton and G. Tough, "Yield and decay of the hydrated electron in proton tracks. A pulse radiolysis study," *Faraday Discussions of the Chemical Society,* vol. 63, pp. 47-54, 1977.

[28] C. Trumbore, D. Short, J. Fanning Jr and J. Olson, "Effects of pulse dose on hydrated electron decay kinetics in the pulse radiolysis of water. A computer modeling study," *The Journal of Physical Chemistry,* vol. 82, no. 26, pp. 2762-2767, 1978.

[29] J. Magee and A. Chatterjee, "A spur unfolding model for the radiolysis of water," *Radiation Physics and Chemistry (1977),* vol. 15, no. 2-3, pp. 125-132, 1980.

[30] D. Swiatla-Wojcik and G. Buxton, "Modeling of radiation spur processes in water at temperatures up to 300. degree," *The Journal of Physical Chemistry,* vol. 99, no. 29, pp. 11464-11471, 1995.

[31] R. Crowell and D. Bartels, "Multiphoton ionization of liquid water with 3.0− 5.0 eV photons," *The Journal of Physical Chemistry,* vol. 100, no. 45, pp. 17940-17949, 1996.

[32] S. Pimblott and J. LaVerne, "Stochastic simulation of the electron radiolysis of water and aqueous solutions," *The Journal of Physical Chemistry A,* vol. 101, no. 33, pp. 5828-5838, 1997.

[33] S. Pimblott and A. Mozumder, "Modeling of physicochemical and chemical processes in the interactions of fast charged particles with matter," in *Charged Particle and Photon Interactions with Matter*, CRC Press, 2003, pp. 85-113.

[34] T. Goulet and J. Jay-Gerin, "Thermalization of subexcitation electrons in solid water," *Radiation research,* vol. 118, no. 1, pp. 46-62, 1989.

[35] J. Meesungnoen, J. Jay-Gerin, A. Filali-Mouhim and S. Mankhetkorn, "Low-energy electron penetration range in liquid water.," *Radiation research,* vol. 158, no. 5, pp. 657-660, 2002.

[36] M. Smith, Computational Study of Low Energy Electrons Through Amorphous Ice and Gaseous Phase Water, The University of Manchester (United Kingdom), 2018.

[37] T. Kai, T. Toigawa, Y. Matsuya, Y. Hirata, T. Tezuka, H. Tsuchida and A. Yokoya, "First-principles simulation of an ejected electron produced by monochromatic deposition energy to water at the femtosecond order," *RSC advances,* vol. 13, no. 46, pp. 32371-32380, 2023.

[38] T. Goulet, J. Jay-Gerin, Y. Frongillo, V. Cobut and M. Fraser, "Rôle des distances de thermalisation des électrons dans la radiolyse de l’eau liquide," *Journal de chimie physique,* vol. 93, pp. 111-116, 1996.

[39] J. Meesungnoen and J. Jay-Gerin, "High-LET radiolysis of liquid water with 1H+, 4He2+, 12C6+, and 20Ne9+ ions: effects of multiple ionization," *The Journal of Physical Chemistry A,* vol. 109, no. 29, pp. 6406-6419, 2005.

[40] R. Signorell, "Electron scattering in liquid water and amorphous ice: a striking resemblance," *Physical Review Letters,* vol. 124, no. 20, p. 205501, 2020.

[41] N. Green and S. Pimblott, "Radiation track structure simulation in a molecular medium," *Research on Chemical Intermediates,* vol. 27, no. 4, pp. 529-538, 2001.

[42] A. D. Bass and L. Sanche, "Charge trapping by H2O condensed onto thin films of Kr and Xe," *The Journal of chemical physics,* vol. 95, no. 4, pp. 2910-2918, 1991.

[43] R. Ogg Jr, "Electronic processes in liquid dielectric media. The properties of metal-ammonia solutions," *Journal of the American Chemical Society,* vol. 68, no. 1, p. 155, 1946.

[44] M. Hilczer and W. Bartczak, "An application of random field theory to analysis of electron trapping sites in disordered media," *The Journal of Physical Chemistry,* vol. 97, no. 2, pp. 508-512, 1993.

[45] M. Hilczer and M. Steblecka, "Primary electron localization in polar liquids: Dependence on matrix and temperature," *Research on chemical*

intermediates, vol. 27, no. 7, pp. 807-822, 2001.

[46] S. Park, W. Narvaez and B. Schwartz, "Ab initio studies of hydrated electron/cation contact pairs: Hydrated electrons simulated with density functional theory are too kosmotropic," *The Journal of Physical Chemistry Letters,* vol. 14, no. 2, pp. 559-566, 2023.
